# Biosimilar recombinant follitropin alfa preparations versus the reference product (Gonal-F®) in couples undergoing assisted reproductive technology treatment: a systematic review and meta-analysis

**DOI:** 10.1186/s12958-021-00727-y

**Published:** 2021-04-02

**Authors:** Su Jen Chua, Ben W. Mol, Salvatore Longobardi, Raoul Orvieto, Christos A. Venetis, Monica Lispi, Ashleigh Storr, Thomas D’Hooghe

**Affiliations:** 1 Austin Health, Heidelberg, VIC 3084 Australia; 2 Department of Obstetrics and Gynecology, University of Monash, Monash, Clayton, Victoria 3168 Australia; 3 Global Clinical Development, Merck Serono S.p.A (an affiliate of Merck KGaA, Darmstadt 64293, Germany), 00176 Rome, Italy; 4 Department of Obstetrics and Gynecology, Chaim Sheba Medical Center, Tel-Hashomer, Ramat Gan, 52621 Israel; 5 The Tarnesby-Tarnowski Chair for Family Planning and Fertility Regulation, Sackler Faculty of Medicine, Tel-Aviv University, Tel Aviv-Yafo, 6997801 Israel; 6 School of Women’s and Children’s Health & Centre for Big Data Research in Health, University of New South Wales, Clayton, Victoria 2052 Australia; 7 IVF Australia, Sydney, NSW 2000 Australia; 8 University of Modena and Reggio Emilia, Modena, MO 41121 Italy; 9 Global Medical Affairs Fertility, Research and Development, Merck KGaA, F135/002, Darmstadt, 64293 Germany; 10 Flinders Fertility, Adelaide, South Australia 5045 Australia; 11 College of Medicine and Public Health, Flinders University, Adelaide, South Australia 5042 Australia; 12 Research Group Reproductive Medicine, Department of Development and Regeneration, Organ Systems, Group Biomedical Sciences, KU Leuven (University of Leuven), Leuven, 3000 Belgium; 13 Department of Obstetrics and Gynecology, Yale University, New Haven, CT 06510 USA

**Keywords:** Biosimilar, Follitropin alfa, IVF, Ovarian stimulation, r-hFSH

## Abstract

**Background:**

Live birth has increasingly been identified as the standard clinical approach to measure the success of medically assisted reproduction (MAR). However, previous analyses comparing biosimilar preparations of follitropin alfa versus the reference product (GONAL-f®, Merck KGaA, Darmstadt, Germany or GONAL-f® RFF; EMD Serono, Inc., Rockland, MA), have had insufficient power to detect differences in clinically meaningful outcomes such as live birth.

**Methods:**

Medline, Embase, the Cochrane Library, Web of Science and clinical trial registries were searched for randomised controlled trials (RCTs) and conference abstracts comparing biosimilar follitropin alfa versus the reference product in controlled ovarian stimulation (COS) cycles published before 31 October 2020. Only studies in humans and publications in English were included. Retrieved studies were screened independently by two authors based on titles and abstracts, and then by full text. Inclusion criteria: RCTs comparing follitropin alfa biosimilar preparations with the reference product in infertile patients of any age, with any type of infertility for any duration, undergoing COS for the purposes of MAR treatment (including frozen cycles). The primary outcome was live birth. Combined data for biosimilar preparations were analysed using a fixed-effects model.

**Results:**

From 292 unique records identified, 17 studies were included in the systematic review, representing five unique RCTs that were included in the meta-analysis. Rates of live birth (RR = 0.83, 95% CI 0.71, 0.97; 4 RCTs, *n* = 1881, I^2^ = 0%), clinical pregnancy (RR = 0.82, 95% CI 0.72, 0.94; 4 RCTs, *n* = 2222, I^2^ = 0%) and ongoing pregnancy (RR = 0.81, 95% CI 0.68, 0.96; 4 RCTs, *n* = 1232, I^2^ = 0%) were significantly lower with biosimilar preparations versus the reference product. Rates of cumulative live birth and cumulative clinical pregnancy were also significantly lower with biosimilars versus the reference product. There was high risk of publication bias.

**Conclusions:**

This meta-analysis included data from RCTs evaluating the efficacy and safety of the biosimilar follitropin alfa preparations and demonstrated lower probability of live birth and pregnancy (ongoing and clinical) in couples treated with biosimilar preparations compared with the reference product. This study provides more insight into the differences between biosimilar r-hFSH preparations and the reference product than previously reported.

**Trial registration:**

Registration number: CRD42019121992.

**Supplementary Information:**

The online version contains supplementary material available at 10.1186/s12958-021-00727-y.

## Background

Exogenous gonadotrophins are used to treat infertility by inducing ovulation or by stimulating multifollicular development in women undergoing medically assisted reproduction (MAR) treatment [1]. The reference product recombinant human follicle-stimulating hormone (r-hFSH, follitropin alfa) was first approved in Europe in 1995 (GONAL-f®, Merck KGaA, Darmstadt, Germany) [2] and in the USA in 1997 (GONAL-f® RFF; EMD Serono, Inc., Rockland, MA) [3] for the induction of multifollicular development in women undergoing MAR treatment. With a predicted 19,245,492 cumulative treatment cycles in women to date (calculated from expected average use per treatment cycle and sales data [4]) and a reported mean live birth rate of 21.7% [5, 6]), more than 4 million babies are estimated to have been born following treatment with GONAL-f®.

Biosimilar preparations, defined as biological medicinal products that contain a version of the active substance of an already authorised original biological medicinal product (reference medicinal product) [7], are also available for follitropin alfa from different marketing authorization holders. According to the European Medicines Agency (EMA), the similarity to the reference medicinal product needs to be established in terms of quality characteristics, biological activity, safety and efficacy based on comprehensive comparability studies before it can be approved for use [7, 8]. Ovaleap® (Theramex, Ireland; launched in 2013) [9, 10] and Bemfola® (Gedeon Richter PLC, Hungary; launched in 2014; also known as Afolia in NCT01687712 and NCT01121666) [11–13] were approved in the EU based on Phase III clinical trials demonstrating non-inferiority to the reference product GONAL-f® for number of oocytes retrieved and comparable safety. Primapur® (iVFarma, LLC, Russia) is due to be launched in Russia but, as there were no requirements for the study primary endpoint set by the Ministry of Health of the Russian Federation, Primapur® was approved based on a clinical study assessing the same endpoint defined by the EMA, which was number of retrieved oocytes [14]. The biosimilar Follitrope® (LG Chem, Ltd., South Korea) has been on the market since 2006 and is available in Asian countries, including China, South Korea, Thailand and Vietnam [15–17]. However, due to the lack of publicly available information regarding the approval of Follitrope®, it is not clear which primary endpoint/clinical outcome was considered for the marketing authorisation approval.

Live birth has been increasingly identified as the standard clinical approach to measure the success of infertility treatment [18–20] and there is increasing consensus that ongoing pregnancy is usually well correlated with live birth [21–23]. Since regulatory approval of biosimilar preparations is governed by a distinct pathway which varies between countries, it is important from a physician and patient perspective to consider all available evidence to evaluate if clinically meaningful differences exist in quality, safety, or efficacy outcomes after use of biosimilar preparations in comparison with the reference product [24–26]. Specifically, for different gonadotrophin preparations used in MAR, the evidence regarding efficacy of biosimilar r-hFSH preparations in terms of live birth or ongoing pregnancy outcomes should be assessed and taken into account, together with the evidence from studies assessing surrogate outcomes [21, 27, 28].

The Phase III clinical trials used for marketing authorisation approval, comparing the biosimilars Bemfola® [11], Ovaleap® [10] and Primapur® [14] with the reference product, also assessed live birth rates as well as ongoing and clinical pregnancy outcomes. However, as these were not primary endpoints of the studies, the analyses were not powered to detect differences in these outcomes [29]. Systematic reviews and meta-analyses are widely accepted methodologies for synthesizing evidence from trials regarding specific research question. Since there is no limitation regarding the type of outcome (primary or secondary) that can be extracted from the original study and analysed, systematic reviews and meta-analyses can be superior to other types of studies in terms of the patient number available for the analysis and the power to detect differences in relevant outcomes [30].

With this in mind, the aim of this meta-analysis was to investigate whether there were any differences in live birth, clinical and ongoing pregnancy rates between biosimilar preparations of follitropin alfa and the reference product using data from published randomised controlled trials (RCTs) and from other credible data sources, in order to provide a comprehensive analysis that takes into account all available evidence.

## Materials and methods

### Study protocol

The study was conducted and reported according to the Preferred Reporting Items for Systematic Reviews and Meta-Analyses (PRISMA) Guidelines (http://www.prisma-statement.org/). A protocol for the systematic review was registered in The International Prospective Register of Systematic Reviews (PROSPERO; CRD42019121992) prior to quantitative analysis.

### Literature searches

Electronic databases (MEDLINE, Embase, Cochrane Library and Web of Science, US Food and Drug Administration [FDA] and EMA) and clinical trial registries (ClinicalTrials.gov and the World Health Organization [WHO] international clinical trial registry platform) were searched for RCTs and conference abstracts comparing biosimilar follitropin alfa preparations with the reference product, published up to 31 October 2020.

The search strategy comprised key words/terms and database-specific indexing terminology on biosimilar preparations of r-hFSH and the reference product (**Supplementary Table** **1****)**. The literature search results were filtered to only include studies in humans, publications in English and to remove any duplicates.

### Study selection

The studies retrieved by the literature search were sequentially screened for inclusion independently by two authors (SJ and AS) based on titles and abstracts and then by full text.

Inclusion criteria, as defined in protocol for systematic review (CRD42019121992), were: RCTs comparing follitropin alfa biosimilar preparations with the reference product in infertile patients of any age, with any type of infertility for any duration, undergoing controlled ovarian stimulation (COS) for the purposes of MAR treatment (including frozen cycles). Only trials in which all aspects of the in vitro fertilisation (IVF) protocols for both treatment arms were the same (except for the use of different r-hFSH preparations: biosimilar preparations versus the reference product), were considered [21]. Crossover trials were included; however, only data for the period of the study before the crossover occurred (e.g. only data from the first cycle) were considered for analysis. RCTs with asymmetric co-interventions between treatment arms, non-randomised studies, cohort studies, case–control studies, case-series, case reports and any studies evaluating drugs for ovarian stimulation other than follitropin alfa biosimilar preparations or the reference product (e.g., follitropin beta, urinary FSH) were excluded. The authors of this review were not blinded to the authors or author institutions of the included RCTs.

### Data collection

The main characteristics of the included studies were independently assessed and extracted by two authors AS and SJ into a predefined standard data extraction form (**Supplementary Tables** **2****and** **3****)** and any disagreement was solved by CAV. Outcomes of (pre-specified) interest were details of treatment protocols used and primary and secondary endpoints of included studies (**Supplementary Table** **4**). In accordance with the Cochrane Handbook for Systematic Reviews of Interventions [30], in the case when the data relevant to the analysis were not available in the published report, attempts were made to contact the authors of the individual studies, or data from other credible sources (e.g., trial registries) were used to extract complete dataset.

### Risk of bias and overall quality of evidence

Risk of bias of individual studies was assessed independently by two reviewers (AS and SJ) using the Cochrane risk of bias tool 2.0 for randomised trials [31]. The overall quality of the evidence was graded according to the Grading of Recommendations Assessment, Development and Evaluation (GRADE) Working Group guidelines [32].

### Endpoints for meta-analysis

The primary endpoint of the meta-analysis was live birth rate per randomised patient. This was defined as the number of deliveries with at least one live birth resulting from one initiated or aspirated treatment cycle, including all cycles in which fresh and/or frozen embryos are transferred, until one delivery with a live birth occurs or until all embryos are used, whichever occurs first. The delivery of a singleton, two or other multiples were registered as one delivery [33]. Only data from the first cycle were used for this endpoint.

Secondary outcomes were clinical pregnancy rate, ongoing pregnancy rate, total dose of gonadotrophins, duration of ovarian stimulation, number of oocytes retrieved per aspirated cycle and number of embryos obtained per aspirated cycle, moderate or severe ovarian hyperstimulation syndrome (OHSS) rate, miscarriage rate, ectopic pregnancy rate, multiple pregnancy rate and immunogenicity (measured by the titres of anti-FSH antibodies). Only moderate or severe OHSS were included in the analysis, as these were considered clinically relevant, and were as defined by the investigators for the eligible individual studies. Clinical pregnancy, miscarriage, ectopic pregnancy and multiple pregnancy were as defined by The International Glossary on Infertility and Fertility Care, 2017 [33]. Ongoing pregnancy was defined as clinical pregnancy at 10–12 weeks. All endpoints were evaluated per randomised patient, with some endpoints (live birth rate, clinical and ongoing pregnancies) also assessed cumulatively. Cumulative live birth was defined as the number of deliveries with at least one live birth, expressed per 100 patients, after a specified time and following all treatments over multiple stimulation cycles.

### Statistical analysis

All data extracted were analysed using the intention-to-treat principle. Pairwise meta-analyses were performed using the fixed-effects model with the Mantel–Haenszel method. In accordance with the Cochrane guidance on Systematic Reviews [34], this review aimed to address the broad question on whether there were any differences in reproductive outcomes after COS with biosimilar follitropin alfa preparations versus the reference product. To this end, data relevant for the experimental intervention (biosimilar preparations) group were combined into a single group and compared with the combined data for the comparator intervention (reference product) group during the analysis. This approach has been widely used in meta-analyses to generate clinical evidence comparing different classes of gonadotrophins used for COS in assisted reproductive technology (ART) treatment [5, 35–41]. Recently, the ESHRE guideline on COS have used these “broad scope” systematic reviews and meta-analyses as first line evidence to elaborate the clinical practice guideline recommendations [42].

The effect size for dichotomous outcomes was presented as relative risk (RR). Uncertainty was expressed using 95% confidence intervals (CI). For continuous data, mean difference was used. Statistical heterogeneity was evaluated with the I^2^ statistic (I^2^ > 50% was indicative of significant heterogeneity). A Funnel plot was used if at least 10 eligible publications were found, to detect publication bias. A sensitivity analysis for the primary endpoint was performed as a random-effects meta-analysis for all comparisons with the exclusion of study with an unclear method of randomisation.

## Results

### Characteristics of the included RCTs

Of the 292 unique publications initially identified, 17 studies were included in the systematic review, which reported data from five unique RCTs (NCT01121666, ISRCTN74772901, NCT01687712, NCT03088137, NCT03506243) (**Supplementary Figure** **1**). The biosimilar preparations investigated were Bemfola® (also known as Afolia; two RCTs) [11–13], Ovaleap® [10], Primapur® [14] and Follitrope® [17]. For the RCT NCT01121666, the data were obtained from the publication by Rettenbacher et al. (2015) [11] and from the EMA Assessment report [12]. For the RCT investigating Ovaleap®, data from the first cycle and subsequent cycles were obtained from separate publications [10, 43]; however, data from the subsequent cycles were not utilised, as all participants crossed over to the exclusive use of Ovaleap® [43].

The main characteristics of the RCTs included in the meta-analysis are summarised in **Supplementary Table** **2****.** Inclusion criteria were generally heterogeneous; however, all five RCTs excluded women with a history of poor response. The RCT investigating Follitrope® excluded patients who had previous history of any type OHSS and the other four studies excluded those who previously had severe OHSS (**Supplementary Table** **3**).

Outcomes assessed in the individual RCTs are summarised in **Supplementary Table** **4**. Four studies reported ongoing pregnancy as defined by clinical pregnancy at 10–12 weeks [10, 11, 14, 17], and three studies reported on clinical pregnancy confirmed by ultrasound at 5–8 weeks [10, 11, 13]. In the Ovaleap® study, four patients who did not achieve pregnancy after first embryo transfer became pregnant after receiving frozen embryos. As a result, in the Ovaleap® study the clinical pregnancy rate was reported separately for only fresh (43/153 in the Ovaleap® group and 52/146 in the GONAL-f® group) or fresh and frozen (46/153 Ovaleap® and 53/146 GONAL-f®) embryo transfer cycles. For the combined analysis, we used the clinical pregnancy rates reported for only fresh embryo transfer cycles. The take-home baby rates, however, were reported for both fresh and frozen cycles combined in the Ovaleap® study; therefore, the combined analysis of live birth rate including only fresh embryo transfer cycles was not possible. Only severe OHSS was reported in two studies, while moderate cases were not reported [14, 17].

The assessment of risk of bias was evaluated as having “some concerns” in two of the RCTs [13, 14]. The method of randomisation and allocation concealment was not reported for one RCT investigating Bemfola®/Afolia (NCT01687712) [13], making it difficult to evaluate the quality of the reported findings; therefore, the evidence for primary and secondary endpoints was graded as moderate for this RCT. The RCT investigating Primapur® (NCT03088137) [14] calculated a power cut-off of 80%, resulting in a smaller sample required to detect equivalence in the number of oocytes retrieved with biosimilar preparations and the reference product. Furthermore, in the RCT evaluating Follitrope® [17], live birth rate was not evaluated, and there was a high attrition after treatment allocation, with only 55% (186/339) of patients allocated to Follitrope® and 71% (79/112) of patients allocated to GONAL-f® receiving an embryo transfer, due to high cancellation rates (44% and 28%, respectively). In addition, per protocol analysis was performed but the deviations from the intended interventions were not reported in the article, and the protocol was not available prior to the study being published, resulting in the study being evaluated as having high risk of bias [17]. Only data from the first cycle of the RCT investigating Ovaleap® [10] were included in the cumulative analysis; therefore, the evidence for cumulative endpoints was graded as low. The corresponding authors of the studies included in this meta-analysis were contacted to obtain additional information on the method of randomisation, IVF protocol and fertility outcomes for Bemfola®/Afolia RCT (NCT01687712) [13] and the immunogenicity data for the RCT investigating Ovaleap® [10]; however, no replies were received. The live birth outcome measurement was deemed as having low risk of bias for four RCTs included in this analysis. Protocol deviations and missing outcomes were addressed by comparing intention-to-treat and as-treated analyses. This was not possible with the Follitrope® study, as only per protocol analysis was reported [17]. Comparison with previously published protocols and trial registries did not reveal reporting bias, with the exception of the study investigating Follitrope® [17]. Given the small number of eligible RCTs, publication bias was not assessed; however, this was likely to be of concern as other biosimilar trials registered in trial registries were detected in the search strategy, which were lacking full publication of results [44–49] (**Supplementary Table** **5**). The authors of these registered trials were also contacted to obtain further information; however, they did not respond.

### Primary endpoint

**Fig. 1:**
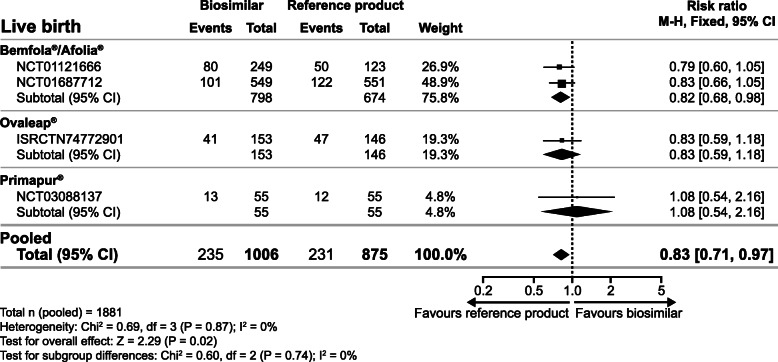
Relative risk for live birth rate with biosimilar preparations of follitropin alfa versus the reference product

Live birth rate was significantly lower with biosimilar preparations (Bemfola®, Ovaleap® and Primapur®) versus the reference product (GONAL-f® or GONAL-f® RFF) (RR 0.83, 95% CI 0.71, 0.97; 4 RCTs, *n* = 1881, I^2^ = 0%, moderate quality evidence, Fig. 1). The sensitivity analysis, which excluded the RCT with an unclear method of randomisation [13], did not alter the effect size, however, it increased the uncertainty around this estimate resulting in a non-statistically significant finding (RR 0.83, 95% CI 0.68, 1.03; 3 RCTs, *n* = 781, I^2^ = 0%, moderate quality evidence, **Supplementary Figure** **2**).

### Secondary endpoints

**Fig. 2:**
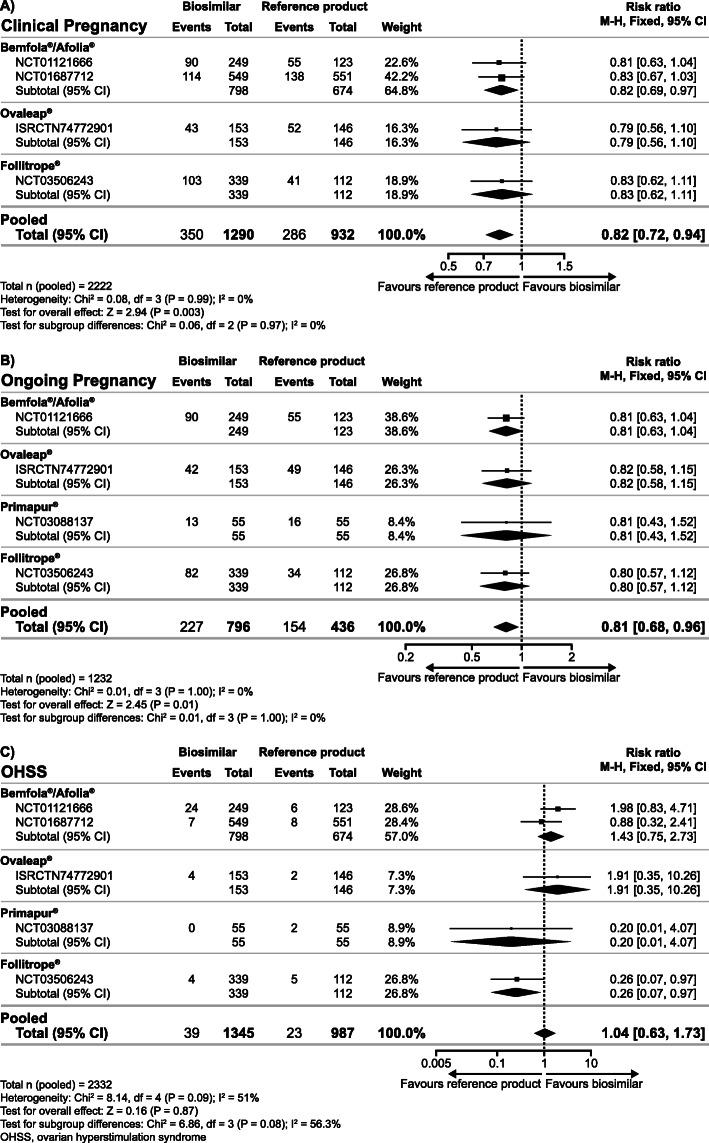
Relative risk for clinical pregnancy rate (**a**), ongoing pregnancy rate (**b**) and ovarian hyperstimulation syndrome (**c**) with biosimilar preparations of follitropin alfa versus the reference product

**Fig. 3:**
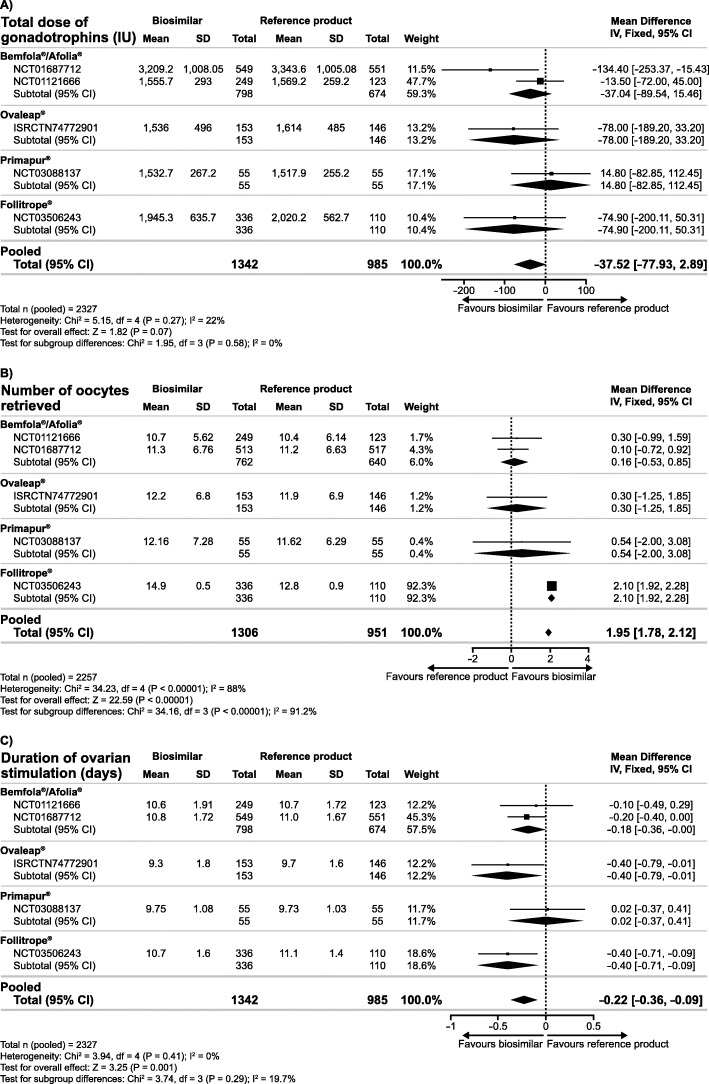
Mean difference in total dose of gonadotrophins (**a**), number of oocytes retrieved (**b**) and duration of ovarian stimulation (**c**) with biosimilar preparations of follitropin alfa versus the reference product

**Fig. 4:**
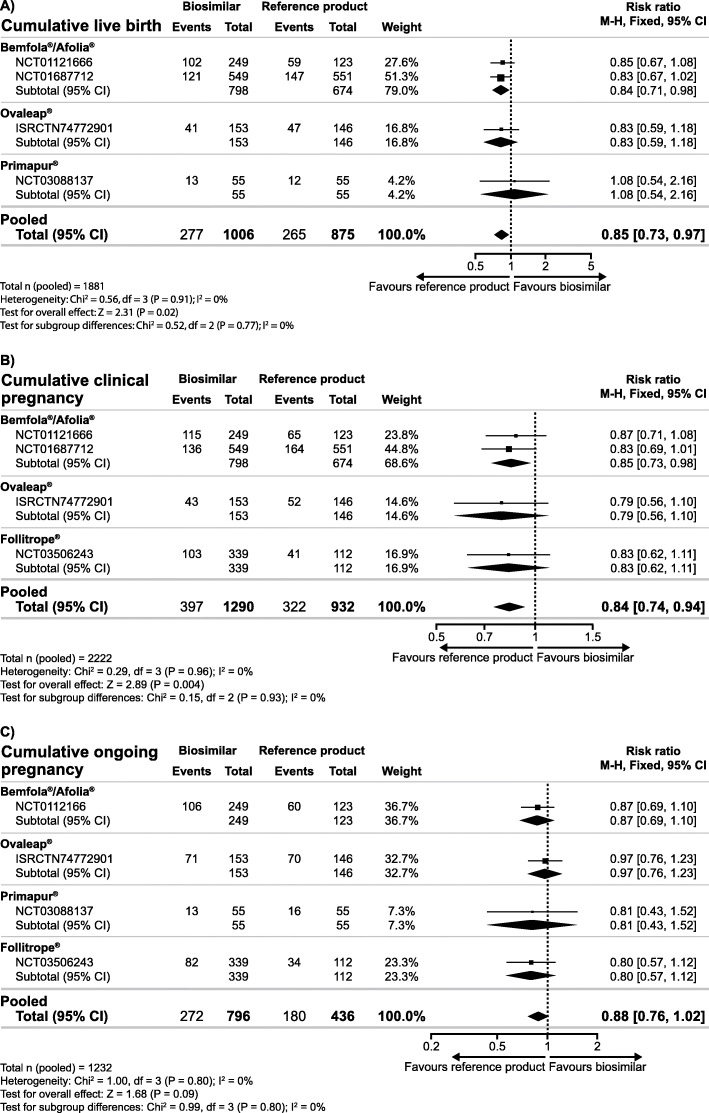
Relative risk for cumulative live birth rate* (**a**), cumulative clinical pregnancy rate (**b**) and cumulative ongoing pregnancy rate (**c**) with biosimilar preparations of follitropin alfa versus the reference product. *For the cumulative live birth, only data from the first cycle could be used for the RCT investigating Ovaleap® as all participants crossed over to the exclusive use of Ovaleap® in subsequent cycles

The secondary analyses of the combined data for biosimilar preparations resulted in significantly lower clinical pregnancy rate and ongoing pregnancy rate observed with biosimilar follitropin alfa preparations compared with the reference product, while the evidence for OHSS rate was inconclusive (Fig. 2). In addition, there was insufficient evidence for a difference in the total dose of gonadotrophins; however, a significantly higher number of oocytes was retrieved and a significantly shorter duration of ovarian stimulation was observed with biosimilar preparations versus the reference product (Fig. 3). Analyses of the cumulative data showed a lower cumulative live birth rate and clinical pregnancy rate observed with biosimilar follitropin alfa preparations versus the reference product, while there was insufficient evidence for a difference in cumulative ongoing pregnancy rate (Fig. 4).

The evidence on ectopic pregnancy rate (RR 1.16, 95% CI 0.39, 3.43; 3 RCTs, *n* = 1509, I^2^ = 0%, moderate quality evidence) and multiple pregnancy rate (RR 1.34, 95% CI 0.61, 2.94; 2 RCTs, *n* = 409, I^2^ = 0%, moderate quality evidence) was inconclusive. Miscarriage rate (foetal loss prior to 22 weeks of gestation [33]) was not available for all studies and was difficult to estimate, as pregnancy up to 22 weeks was not reported in all of the studies. Immunogenicity and the number of embryos obtained were not evaluated in this meta-analysis, owing to a lack of data or heterogeneity in the methods used to assess these outcomes.

## Discussion

This meta-analysis included data from the Phase III clinical trials evaluating respective biosimilar follitropin alfa preparations in the EU, USA, Russia and China, and demonstrated lower probability of live birth, ongoing and clinical pregnancy in couples treated with biosimilar preparations compared with the reference product. Available data from up to three cycles allowed the evaluation of cumulative outcomes, which showed lower cumulative live birth and clinical pregnancy rates for biosimilar preparations versus the reference product. Safety data suggested that biosimilar preparations had a similar risk of OHSS, ectopic pregnancy and multiple pregnancy compared with the reference product.

Our findings show that although the number of oocytes retrieved was slightly higher (one more egg in all studies, except in the Follitrope® study reporting two more eggs), lower pregnancy rates were reported with biosimilar preparations versus the reference product. To investigate this further, we conducted an additional analysis which excluded the Follitrope® study [17], which was identified as having a high risk of bias. The exclusion of the Folitrope® study from the analysis resulted in insufficient evidence for a difference in the number of oocytes retrieved with GONAL-f® versus biosimilars (mean difference 0.20, 95% CI -0.41, 0.81; 4 RCTs; *n* = 1881; I^2^ = 0%, moderate quality evidence). This finding should therefore be interpreted with caution. Furthermore, the mean total number of eggs varied between 10 and 15 in the five RCTs considered (Fig. 3b), which are normal numbers expected from a population with a normal ovarian reserve receiving a 150 – 225 IU r-hFSH starting dose [2, 50–52]. Therefore, this observation is not in conflict with current opinion that the number of oocytes retrieved positively correlates with downstream fertility treatment outcomes, including pregnancy and live birth [50–58].

It is common to see comparability studies for infertility medications adopting the number of retrieved oocytes as a primary (surrogate) endpoint, as this avoids the impact of confounding factors that might not be attributable to these medications, and it is also more economical [59]. Nonetheless, there are several other factors that can have an impact on the success of IVF treatment, such as the quality of the oocytes, embryos [60] and the endometrium [61]. Previous studies have shown that there are differences in biological activity, composition of isoforms, glycosylation patterns and clearance rates observed between different preparations of r-hFSH [8, 29]. This may affect their mode of action on FSH receptors in the ovary and therefore have an impact on the quality of oocytes [8, 29]. It is also important to note that the assessment of oocyte and embryo quality is often heterogeneous, with different oocyte and embryo grading systems used across different clinics, which makes inter-laboratory and inter-study comparisons extremely difficult [62]. The implementation of a unified objective approach to assess the quality of oocytes and embryos across different clinics is therefore required before fair comparisons can be made to evaluate the effectiveness of different treatment options.

Choosing between biosimilar preparations and the reference product can prove challenging. We believe that the decision regarding whether to use biosimilar preparations or the reference product should be reserved to the treating physician, based on clinical efficacy and safety characteristics, real-world effectiveness, cost-effectiveness studies and patient preference [8]. There is still a debate with regard to using surrogate endpoints to measure the success of infertility treatment, due to the fact that they do not capture the effect of the treatment on clinically relevant outcomes [63]. Although one treatment option can appear to be equally effective in terms of midway (upstream) outcomes — such as number of oocytes retrieved — the ultimate goal of fertility treatment is pregnancy leading to live birth. While the studies evaluating midway (upstream) fertility treatment outcomes to compare treatment options are scientifically valid, they fail to answer the ultimate question of whether the treatments are comparable in terms of live birth. It has been confirmed in the ESHRE Guideline for Ovarian Stimulation in IVF/ICSI and in the International Committee for Monitoring Assisted Reproductive Technology (ICMART) revised glossary that the most relevant outcomes of infertility treatment are live birth rate and cumulative live birth rate [33, 42]. Studies should therefore aim to evaluate these endpoints in order to measure the comparability between r-hFSH biosimilar preparations and the reference product.

A recent meta-analysis by Budani et al. [64] evaluated a range of efficacy outcomes with Bemfola®, Ovaleap® and Primapur® biosimilar preparations (*n* = 457 women in total) versus the reference product GONAL-f (*n* = 324), based on the data from the same three RCTs that were included in our meta-analysis [10, 11, 14]. Although a significantly lower clinical pregnancy rate was seen with the biosimilar preparations compared with the reference product (odds ratio 0.71, 95% CI 0.52, 0.97), no difference was reported for take-home baby rate or other outcomes of interest [64]. This may be due to insufficient power to detect differences in these outcomes, as the analysis by Budani et al. was restricted to only three published RCTs, thus limiting the number of patients included in the analysis.

Compared to the meta-analysis by Budani et al. [64], our meta-analysis included a higher number of patients, which allowed evaluation of treatment effects on the outcome of interest with a greater statistical power. In addition, we have included the data reported in the Phase III trial of Follitrope® versus GONAL-f®, which was not included in the analysis by Budani et al. Although Follitrope® was not defined as a biosimilar to follitropin alfa in the publication [17]; after appropriate assessment of local registration procedure, it was confirmed that Follitrope® is a biosimilar of GONAL-f® [15–17, 65]. Furthermore, in accordance with Cochrane guidance on Systematic Reviews, we have considered data from all credible sources related to the selected RCTs, including clinical trial databases and regulatory documents [30]. This extended dataset enabled us to report on cumulative endpoints, which would not be possible taking into account only the data reported in RCT publications, which, by design, are often limited to a pre-specified and narrow timeframe.

The populations assessed in the individual RCTs included in our meta-analysis mostly consisted of young, good-prognosis couples with normal response to gonadotrophin treatment, which poorly reflects the overall population of patients that are actually treated at infertility clinics. To provide the full picture in terms of comparability of r-hFSH biosimilars to the reference product, we recommend that further RCTs and also real-world data analyses should be conducted to assess other patient populations that are treated during routine clinical practice. This includes older patients, those with a poor or high response to ovarian stimulation and patients with repeated IVF failures. Such studies should also aim to compare cumulative outcomes, to account for the need for multiple ART cycles and the potential differences between patients undergoing their first cycle and those treated in subsequent cycles after they did not have a pregnancy leading to live birth in previous attempts [19].

More studies are required to assess the comparative effectiveness of biosimilar preparations in terms of clinically meaningful MAR outcomes in a real-world setting. It has been argued that analysis of data from large observational databases can be complementary to data analysis from RCTs when investigating and comparing the effectiveness of different MAR treatment options. Real-world studies can include a large number of patients and treatment cycles, representing the reality of clinical practice, and need to take into account the baseline and treatment confounders associated with this heterogeneous population, to evaluate if clinically meaningful differences in live birth rates exist between different MAR treatment options [66–69]. Furthermore, RCTs often have a limited follow-up time, therefore making assessment of outcomes, such as live birth, challenging. In contrast, real-world data studies can allow a longer follow-up, including assessment of live birth data, as well as obstetrical and neonatal data, thus providing additional information about long-term effectiveness of a medication. Finally, as there are differences in the costs associated with biosimilar follitropin alfa preparations versus the reference product used during ovarian stimulation, cost-effectiveness studies should be conducted in order to make informed decisions from a health economics perspective.

One of the strengths of our meta-analysis was that the data analysed for the primary outcome collectively comprised 1881 patients, which is a sufficient number for hypothesis generation. To increase the number of patients available for the quantitative analysis, we have combined the data for the respective biosimilar preparations in the experimental intervention group for comparison with the reference product, in accordance to Cochrane Handbook for Systematic Reviews of Interventions [34] and other systematic reviews/meta-analyses where outcomes were not reported according to individual gonadotrophin preparations [5, 35–41]. The protocol for this systematic review, including the study objectives, pre-defined inclusion and exclusion criteria and planned analyses, was registered in PROSPERO prior to literature search being conducted. As such, the design of quantitative analysis was not, a priori, affected by the results of individual trials included in this study. Finally, there was generally a low level of heterogeneity among the studies for most of the outcomes.

This study had some inherent limitations. Only a small number of studies were included in this meta-analysis, and most participants (*n* = 1100) originated from a single large RCT investigating Bemfola®/Afolia [13], which was evaluated as having some concerns for the risk of bias as no information was reported on the methods used for randomisation and allocation concealment. However, in our opinion, as this Bemfola®/Afolia RCT was conducted for marketing authorisation approval purposes usually associated with stringent quality criteria, this clinical trial was likely of a high quality, with a detailed study protocol and data analysis plan, despite the lack of publicly available information on randomisation and allocation concealment. Four frozen embryo cycles originating from one study [10] were included in the combined analysis for live birth, which may have affected the comparison between biosimilars and the reference product for this outcome. Furthermore, the included studies compared outcomes for only four biosimilar preparations with the reference product. There are several other biosimilar gonadotrophin preparations available on the market; however, the data either remain unpublished or the studies identified did not assess the fertility outcomes of interest (**Supplementary Table** **5**) and attempts to obtain more information from the authors were unsuccessful. This indicates a need for more head-to-head studies to evaluate the possible differences in outcomes among all follitropin alfa preparations. The evidence for cumulative data was judged as low quality, as crossover occurred after the first cycle in the study investigating Ovaleap®; therefore, only data from the first cycle were included from this study. In addition, patients often discontinued ART treatment during subsequent cycles due to non-medical reasons (e.g. funding, burden of treatment), and while an attempt was made to accommodate this by using an intention-to-treat analysis, this was not possible for the Follitrope® study, and there is still risk of bias in the other studies as well. Moreover, women with severe OHSS were excluded from additional cycles, and women who did not achieve live birth in the first cycle were offered subsequent ART treatment cycles.

## Conclusions

This meta-analysis suggests that treatment with biosimilar preparations of follitropin alfa is likely to result in lower probability of live birth, clinical and ongoing pregnancy compared with the reference product. Safety data showed that biosimilar preparations carried a similar risk of OHSS, ectopic pregnancy and multiple pregnancy compared with the reference product. More head-to-head RCTs as well as real-world studies are required to ascertain clinically relevant fertility outcomes, including cumulative pregnancy and live birth rates.

## Supplementary Information

**Additional file 1.** Supplementary Figure 1. Flow chart of study selection. Supplementary Figure 2. Relative risk for live birth rate with biosimilar preparations of follitropin alfa versus reference product (sensitivity analysis excluding the study with an unclear method of randomisation). Supplementary Table 1. Search strategy. Supplementary Table 2. Main characteristics of the randomised controlled trials included in the meta-analysis. Supplementary Table 3. Population characteristics, details of assisted reproductive technology treatment protocol used, outcomes evaluated and adjustment for confounders of the randomised controlled trials included in the meta-analysis. Supplementary Table 4. Outcomes of the randomised controlled trials included in the meta-analysis. Supplementary Table 5. Summary of the randomised controlled trials detected by search strategy without fertility outcomes.

## Data Availability

Any requests for data by qualified scientific and medical researchers for legitimate research purposes will be subject to Merck KGaA’s Data Sharing Policy. All requests should be submitted in writing to Merck KGaA’s data sharing portal https://www.merckgroup.com/en/research/our-approach-to-research-and-development/healthcare/clinical-trials/commitment-responsible-data-sharing.html. When Merck KGaA has a co-research, co-development, or co-marketing or co-promotion agreement, or when the product has been out-licensed, the responsibility for disclosure might be dependent on the agreement between parties. Under these circumstances, Merck KGaA will endeavour to gain agreement to share data in response to requests.

## Abbreviations

ART: Assisted reproductive technology
CI: Confidence intervals
COS: Controlled Ovarian Stimulation
EMA: European Medicines Agency
FDA: Food and Drug Administration
FSH: Follicle-stimulating hormone
GRADE: Grading of Recommendations Assessment, Development and Evaluation
ICMART: International Committee for Monitoring Assisted Reproductive Technology
IVF: In vitro fertilisation
MAR: Medically assisted reproduction
OHSS: Ovarian hyperstimulation syndrome
PRISMA: Preferred Reporting Items for Systematic Reviews and Meta-Analyses
RCT: Randomised controlled trial
r-hFSH: Recombinant human follicle-stimulating hormone
RR: Relative risk
WHO: World Health Organization

## References

[1] Lunenfeld B, Bilger W, Longobardi S, Alam V, D'Hooghe T, Sunkara SK (2019). The development of gonadotropins for clinical use in the treatment of infertility. Front Endocrinol.

[2] European Medicines Agency (2010). GONAL-f (follitropin alfa): summary of product characteristics.

[3] Food and Drug Administration (2013). GONAL-F® RFF* REDI-JECT™ (follitropin alfa): prescribing information.

[4] Velthuis E, Hubbard J, Longobardi S, D'Hooghe T (2020). The frequency of ovarian Hyperstimulation syndrome and thromboembolism with originator recombinant human Follitropin Alfa (GONAL-f) for medically assisted reproduction: a systematic review. Adv Ther.

[5] Al-Inany HG, Abou-Setta AM, Aboulghar MA, Mansour RT, Serour GI (2008). Efficacy and safety of human menopausal gonadotrophins versus recombinant FSH: a meta-analysis. Reprod BioMed Online.

[6] Coomarasamy A, Afnan M, Cheema D, van der Veen F, Bossuyt PM, van Wely M (2008). Urinary hMG versus recombinant FSH for controlled ovarian hyperstimulation following an agonist long down-regulation protocol in IVF or ICSI treatment: a systematic review and meta-analysis. Hum Reprod.

[7] European Medicines Agency (2014). Guideline on similar biological medicinal products containing biotechnology-derived proteins as active substance: non-clinical and clinical issues.

[8] Orvieto R, Seifer DB (2016). Biosimilar FSH preparations- are they identical twins or just siblings?. Reprod Biol Endocrinol.

[9] European Medicines Agency (2013). EPAR summary for the public: Ovaleap (follitropin alfa).

[10] Strowitzki T, Kuczynski W, Mueller A, Bias P (2016). Randomized, active-controlled, comparative phase 3 efficacy and safety equivalence trial of Ovaleap(R) (recombinant human follicle-stimulating hormone) in infertile women using assisted reproduction technology (ART). Reprod Biol Endocrinol.

[11] Rettenbacher M, Andersen AN, Garcia-Velasco JA, Sator M, Barri P, Lindenberg S, van der Ven K, Khalaf Y, Bentin-Ley U, Obruca A (2015). A multi-Centre phase 3 study comparing efficacy and safety of Bemfola((R)) versus Gonal-f((R)) in women undergoing ovarian stimulation for IVF. Reprod BioMed Online.

[12] European Medicines Agency (2014). Assessment report: Bemfola.

[13] Fertility Biotech AG (2012). Phase III Study Comparing Efficacy and Safety of AFOLIA vs Gonal-f® RFF in Women (35 to 42) Undergoing IVF. U.S. National Library of Medicine.

[14] Barakhoeva Z, Vovk L, Fetisova Y, Marilova N, Ovchinnikova M, Tischenko M, Scherbatyuk Y, Kolotovkina A, Miskun A, Kasyanova G (2019). A multicenter, randomized, phase III study comparing the efficacy and safety of follitropin alpha biosimilar and the original follitropin alpha. Eur J Obstet Gynecol Reprod Biol.

[15] Asian-Pacific Biotech News (2008). An Exclusive on LG Life Sciences.

[16] Drugs.com: Follitrope. Available at: https://www.drugs.com/international/follitrope.html. Accessed 17 Nov 2020

[17] Hu L, Zhang S, Quan S, Lv J, Qian W, Huang Y, Lu W, Sun Y (2020). Efficacy and safety of recombinant human follicle-stimulating hormone in patients undergoing in vitro fertilization-embryo transfer. Aging (Albany NY).

[18] Braam SC, de Bruin JP, Buisman E, Brandes M, Nelen W, Smeenk JMJ, van der Steeg JW, Mol BWJ, Hamilton C (2018). Treatment strategies and cumulative live birth rates in WHO-II ovulation disorders. Eur J Obstet Gynecol Reprod Biol.

[19] Malizia BA, Hacker MR, Penzias AS (2009). Cumulative live-birth rates after in vitro fertilization. N Engl J Med.

[20] Germond M, Urner F, Chanson A, Primi MP, Wirthner D, Senn A (2004). What is the most relevant standard of success in assisted reproduction?: the cumulated singleton/twin delivery rates per oocyte pick-up: the CUSIDERA and CUTWIDERA. Hum Reprod.

[21] Mol BW, Bossuyt PM, Sunkara SK, Garcia Velasco JA, Venetis C, Sakkas D, Lundin K, Simón C, Taylor HS, Wan R (2018). Personalized ovarian stimulation for assisted reproductive technology: study design considerations to move from hype to added value for patients. Fertil Steril.

[22] Braakhekke M, Kamphuis EI, van Rumste MM, Mol F, van der Veen F, Mol BW (2014). How are neonatal and maternal outcomes reported in randomised controlled trials (RCTs) in reproductive medicine?. Hum Reprod.

[23] Clarke JF, van Rumste MM, Farquhar CM, Johnson NP, Mol BW, Herbison P (2010). Measuring outcomes in fertility trials: can we rely on clinical pregnancy rates?. Fertil Steril.

[24] Wolff-Holz E, Tiitso K, Vleminckx C, Weise M (2019). Evolution of the EU biosimilar framework: past and future. BioDrugs.

[25] Biosimilars in the EU (2017). Information guide for healthcare professionals prepared jointly by the European Medicines Agency and the European Commission.

[26] US Food and Drug Administration (2015). Scientific considerations in demonstrating biosimilarity to a reference product.

[27] Vail A, Gardener E (2003). Common statistical errors in the design and analysis of subfertility trials. Hum Reprod.

[28] Improving the Reporting of Clinical Trials of Infertility Treatments (IMPRINT) (2014). modifying the CONSORT statement. Fertil Steril.

[29] Bergandi L, Canosa S, Carosso AR, Paschero C, Gennarelli G, Silvagno F, Benedetto C, Revelli A. Human recombinant FSH and its Biosimilars: clinical efficacy, safety, and cost-effectiveness in controlled ovarian stimulation for in vitro fertilization. Pharmaceuticals (Basel). 2020;13:1-20. 10.3390/ph13070136 PMC7407829 32605133

[30] Li T, JPT H, Deeks JJ, JPT H, Thomas J, Chandler J, Cumpston M, Li T, Page MJ, Welch VA (2019). Section 5.2: sources of data in chapter 5: collecting data. Cochrane handbook for systematic reviews of interventions version 6.0 (updated July 2019). Cochrane.

[31] Sterne JAC, Savović J, Page MJ, Elbers RG, Blencowe NS, Boutron I, Cates CJ, Cheng HY, Corbett MS, Eldridge SM (2019). RoB 2: a revised tool for assessing risk of bias in randomised trials. BMJ.

[32] GRADEpro GDT (2015). GRADEpro Guideline Development Tool [Software]. McMaster University.

[33] Zegers-Hochschild F, Adamson GD, Dyer S, Racowsky C, de Mouzon J, Sokol R, Rienzi L, Sunde A, Schmidt L, Cooke ID (2017). The international glossary on infertility and Fertility care, 2017. Fertil Steril.

[34] Thomas J, Kneale D, JE MK, Brennan SE, Bhaumik S, JPT H, Thomas J, Chandler J, Cumpston M, Li T, Page MJ, Welch VA (2020). Section 2.3.1: broad versus narrow reviews in chapter 2: determining the scope of the review and the questions it will address. Cochrane handbook for systematic reviews of interventions version 6.1 (updated September 2020). Cochrane.

[35] Al-Inany H, Aboulghar M, Mansour R, Serour G (2003). Meta-analysis of recombinant versus urinary-derived FSH: an update. Hum Reprod.

[36] Al-Inany HG, Youssef MA, Ayeleke RO, Brown J, Lam WS, Broekmans FJ (2016). Gonadotrophin-releasing hormone antagonists for assisted reproductive technology. Cochrane Database Syst Rev.

[37] Bordewijk EM, Mol F, van der Veen F, Van Wely M (2019). Required amount of rFSH, HP-hMG and HP-FSH to reach a live birth: a systematic review and meta-analysis. Hum Reprod Open.

[38] van Wely M, Kwan I, Burt AL, Thomas J, Vail A, Van der Veen F, Al-Inany HG (2011). Recombinant versus urinary gonadotrophin for ovarian stimulation in assisted reproductive technology cycles. Cochrane Database Syst Rev.

[39] Wang R, Lin S, Wang Y, Qian W, Zhou L (2017). Comparisons of GnRH antagonist protocol versus GnRH agonist long protocol in patients with normal ovarian reserve: a systematic review and meta-analysis. PLoS One.

[40] Weiss NS, Nahuis M, Bayram N, Mol BW, Van der Veen F, van Wely M. Gonadotrophins for ovulation induction in women with polycystic ovarian syndrome. Cochrane Database Syst Rev. 2015:Cd010290. 10.1002/14651858.CD010290.pub2 26350625

[41] Youssef MA, Van der Veen F, Al-Inany HG, Mochtar MH, Griesinger G, Nagi Mohesen M, Aboulfoutouh I, van Wely M. Gonadotropin-releasing hormone agonist versus HCG for oocyte triggering in antagonist-assisted reproductive technology. Cochrane Database Syst Rev. 2014:Cd008046. 10.1002/14651858.CD008046.pub4 PMC10767297 25358904

[42] European Society of Human Reproduction and Embryology (ESHRE) Reproductive Endocrinology Guideline Group (2019). Ovarian Stimulation for IVF/ICSI.

[43] Strowitzki T, Kuczynski W, Mueller A, Bias P (2016). Safety and efficacy of Ovaleap® (recombinant human follicle-stimulating hormone) for up to 3 cycles in infertile women using assisted reproductive technology: a phase 3 open-label follow-up to Main study. Reprod Biol Endocrinol.

[44] LG Life Sciences India Pvt Ltd (2013). A clinical trial to see the effects, safety and patient compliance of two drugs Newmon-RTM pre filled syringe and Gonal-F® pen in infertile women undergoing IVF treatment. WHO.

[45] Bharat Serums and Vaccines Ltd (2016). Effect of Foligraf™ and Gonal-F® in assisted reproductive technology. WHO.

[46] Cadila Healthcare Limited (2016). Phase III Study to Evaluate the Efficacy and Safety of Recombinant Human FSH of Cadila Healthcare Limited, India as compared to Gonal-F Administered Subcutaneously in Female Patients Undergoing Assisted Reproductive Technology. WHO.

[47] Watson Laboratories Inc.: Clinical study to compare the safety and effectiveness of Actavis rhFSH (the medicine being developed) with GONAL-f (an approved medicine) in Stimulating Multiple Follicles (a woman's eggs or ova) in Women Participating in an Assisted Reproductive Technology Program (such as *in vitro* fertilisation, 'IVF'). WHO; 2014. Report no.: EUCTR2013-003788-67-BE. Available at: https://apps.who.int/trialsearch/Trial2.aspx? TrialID=EUCTR2013-003788-67-AT. Accessed October 2020.

[48] Teymouri FA (2016). Comparison of efficacy Cinnal-F and Gonal-F on infertility treatment (ICSI). Iranian Registry of Clinical Trials.

[49] Gema Biotech SA (2015). A Randomized, Multicentre, Open Label, Evaluator Blinded Study to Evaluate Safety and Efficacy of Folitime® of Gemabiotech S.A., Versus Gonal-f® of Merck Serono, in Patients With Infertility Undergoing ART. U.S. National Library of Medicine.

[50] Sunkara SK, Rittenberg V, Raine-Fenning N, Bhattacharya S, Zamora J, Coomarasamy A (2011). Association between the number of eggs and live birth in IVF treatment: an analysis of 400 135 treatment cycles. Hum Reprod.

[51] Drakopoulos P, Blockeel C, Stoop D, Camus M, de Vos M, Tournaye H, Polyzos NP (2016). Conventional ovarian stimulation and single embryo transfer for IVF/ICSI. How many oocytes do we need to maximize cumulative live birth rates after utilization of all fresh and frozen embryos?. Hum Reprod.

[52] Ji J, Liu Y, Tong XH, Luo L, Ma J, Chen Z (2013). The optimum number of oocytes in IVF treatment: an analysis of 2455 cycles in China. Hum Reprod.

[53] Magnusson Å, Källen K, Thurin-Kjellberg A, Bergh C (2018). The number of oocytes retrieved during IVF: a balance between efficacy and safety. Hum Reprod.

[54] Polyzos NP, Sunkara SK (2015). Sub-optimal responders following controlled ovarian stimulation: an overlooked group?. Hum Reprod.

[55] Toftager M, Bogstad J, Løssl K, Prætorius L, Zedeler A, Bryndorf T, Nilas L, Pinborg A (2017). Cumulative live birth rates after one ART cycle including all subsequent frozen-thaw cycles in 1050 women: secondary outcome of an RCT comparing GnRH-antagonist and GnRH-agonist protocols. Hum Reprod.

[56] Vaughan DA, Leung A, Resetkova N, Ruthazer R, Penzias AS, Sakkas D, Alper MM (2017). How many oocytes are optimal to achieve multiple live births with one stimulation cycle? The one-and-done approach. Fertil Steril.

[57] Verberg MF, Eijkemans MJ, Macklon NS, Heijnen EM, Baart EB, Hohmann FP, Fauser BC, Broekmans FJ (2009). The clinical significance of the retrieval of a low number of oocytes following mild ovarian stimulation for IVF: a meta-analysis. Hum Reprod Update.

[58] van Loendersloot LL, van Wely M, Limpens J, Bossuyt PM, Repping S, van der Veen F (2010). Predictive factors in in vitro fertilization (IVF): a systematic review and meta-analysis. Hum Reprod Update.

[59] de Mora F, Fauser BCJM (2017). Biosimilars to recombinant human FSH medicines: comparable efficacy and safety to the original biologic. Reprod BioMed Online.

[60] Zhao Y-Y, Yu Y, Zhang X-W (2018). Overall blastocyst quality, Trophectoderm Grade, and inner cell mass Grade predict pregnancy outcome in Euploid blastocyst transfer cycles. Chin Med J.

[61] Gallos ID, Khairy M, Chu J, Rajkhowa M, Tobias A, Campbell A, Dowell K, Fishel S, Coomarasamy A (2018). Optimal endometrial thickness to maximize live births and minimize pregnancy losses: analysis of 25,767 fresh embryo transfers. Reprod BioMed Online.

[62] Alpha Scientists in Reproductive Medicine and ESHRE Special Interest Group of Embryology (2011). The Istanbul consensus workshop on embryo assessment: proceedings of an expert meeting. Hum Reprod.

[63] Barnhart KT (2014). Live birth is the correct outcome for clinical trials evaluating therapy for the infertile couple. Fertil Steril.

[64] Budani MC, Fensore S, Di Marzio M, Tiboni GM. Efficacy and safety of follitropin alpha biosimilars compared to their reference product: a meta-analysis. Gynecol Endocrinol. 2020:1–9. 10.1080/09513590.2020.1792437 32654532

[65] Biosimilar medicines: Overview [https://www.ema.europa.eu/en/human-regulatory/overview/biosimilar-medicines-overview]. Accessed 17 Nov 2020

[66] Wilkinson J, Brison DR, Duffy JMN, Farquhar CM, Lensen S, Mastenbroek S, van Wely M, Vail A (2019). Don’t abandon RCTs in IVF. We don’t even understand them. Hum Reprod.

[67] Harari S. Randomised controlled trials and real-life studies: two answers for one question. Eur Respir Rev. 2018;27:180080. 10.1183/16000617.0080-2018 PMC9488996 30257909

[68] Hershkop E, Segal L, Fainaru O, Kol S (2017). 'Model' versus 'everyday' patients: can randomized controlled trial data really be applied to the clinic?. Reprod BioMed Online.

[69] Garrison LP, Neumann PJ, Erickson P, Marshall D, Mullins CD (2007). Using real-world data for coverage and payment decisions: the ISPOR real-world data task force report. Value Health.

