# Correction: Biosimilar recombinant follitropin alfa preparations versus the reference product (Gonal-F®) in couples undergoing assisted reproductive technology treatment: a systematic review and meta-analysis

**DOI:** 10.1186/s12958-023-01114-5

**Published:** 2023-07-26

**Authors:** Christos A. Venetis, Christoph Helwig, Ben W. Mol, Su Jen Chua, Salvatore Longobardi, Raoul Orvieto, Monica Lispi, Ashleigh Storr, Thomas D’Hooghe

**Affiliations:** 1 Unit for Human Reproduction, 1st Dept of OB/Gyn, Faculty of Medicine, School of Health Sciences, Aristotle University of Thessaloniki, Thessaloniki, Greece; 2 Centre for Big Data Research in Health, University of New South Wales, Kensington Campus, New South Wales, Australia; 3 IVF Australia, Sydney, NSW 2000 Australia; 4 Global Biostatistics, Merck KGaA, Darmstadt, Germany; 5 Department of Obstetrics and Gynecology, University of Monash, Monash, Clayton, Victoria 3168 Australia; 6 Alfred Health, Melbourne, VIC 3004 Australia; 7 Global Clinical Development, Merck Serono S.p.A (an affiliate of Merck KGaA, Darmstadt 64293, Germany), Rome, 00176 Italy; 8 Department of Obstetrics and Gynecology, Chaim Sheba Medical Center, Tel-Hashomer, Ramat Gan, 52621 Israel; 9 The Tarnesby-Tarnowski Chair for Family Planning and Fertility Regulation, Sackler Faculty of Medicine, Tel-Aviv University, Tel Aviv-Yafo, 6997801 Israel; 10 University of Modena and Reggio Emilia, Modena, MO 41121 Italy; 11 GlobalMedical Affairs Fertility, Research and Development, Merck KGaA, F135/002, Darmstadt, 64293 Germany; 12 Flinders Fertility, Adelaide, South Australia 5045 Australia; 13 College of Medicine and Public Health, Flinders University, Adelaide, South Australia 5042 Australia; 14 Research Group Reproductive Medicine, Department of Development and Regeneration, Organ Systems, Group Biomedical Sciences, KU Leuven (University of Leuven), Leuven, 3000 Belgium; 15 Department of Obstetrics and Gynecology, Yale University, New Haven, CT 06510 USA

**Correction: Reprod Biol Endocrinol 19, 51 (2021)**

**https://doi.org/10.1186/s12958-021-00727-y**

Following publication of the original article [1], the authors would like readers to acknowledge the below corrections, based on inconsistencies in one of the articles included in the meta-analysis.

The secondary outcomes of the meta-analysis presented by Chua et al. [1] included the number of oocytes retrieved per aspirated cycle. This calculation included data from a randomized controlled trial (RCT) by Hu et al. (NCT03506243) comparing Follitrope® (LG Chem, Ltd., South Korea) and GONAL-f® (Merck Healthcare KGaA, Darmstadt, Germany) [2]. This was based on the mean ± standard deviation (SD) number of oocytes retrieved, which was reported by Hu et al. in Table 2 of their publication (14.9 ± 0.5 for Follitrope [*n* = 336] and 12.8 ± 0.9 for GONAL-f® [*n* = 110]) [2]. Owing to the very small variability in the number of oocytes retrieved, as reported in this RCT, this RCT was given a high weighting (> 90%) compared with the other four RCTs included in this estimation (see Fig. 3B in our article [1]).

We now have reason to believe that the oocyte number reported in Table 2 of the article by Hu et al. was actually reported as the least square mean ± standard error (SE), rather than mean ± SD, as stated in the table footnote. Our premise is supported by the following points.

The primary endpoint reported by Hu et al. was the number of oocytes retrieved [2]. The Materials and Methods (Sample size calculation and statistical analysis section) state that, ‘to compare the primary efficacy endpoints, an analysis of variance model was used, with treatment group, site, and treatment as fixed categorical effects’. In addition, ‘for all efficacy outcomes, summary statistics (mean, SD, median, minimum, maximum, quartile) for each group were presented’. Therefore, the analysis of the primary endpoint was expected to be reported as least square mean ± standard error.

In the Results section (Efficacy; Primary outcome section) the least square mean (± SD) number of oocytes is reported as 14.9 (± 0.5; median [range]: 14 [1 to 41]) in the Follitrope group, and 12.8 (± 0.9; median [range]: 13 [3 to 33]) in the GONAL-F group, which are the same numbers as those reported in Table 2 as the mean (± SD) number of oocytes retrieved [2].

The precision values reported in Table 2 are almost double in the smaller group (GONAL-f [*n* = 110]) compared with the larger group (Follitrope [*n* = 336]) [2]. This is usually a characteristic of the standard error (SE), which is dependent on the sample size; therefore, smaller groups would lead to higher SEs (as in this case), while the SD is not dependent on sample size.

When comparing the SD values reported in Table 2 [2] with those from the other four RCTs included our meta-analysis (NCT01121666, NCT01687712, ISRCTN74772901 and NCT03088137) [1], the values are unusually small and inconsistent with the range of SDs reported for the other four RCTs.

In Supplementary Table 1, efficacy outcomes are reported by age subgroups as well as for the total analysis set [2]. The mean ± SD total number of oocytes retrieved reported in this table are 15.4 ± 7.5 for Follitrope and 13.9 ± 6.4 for GONAL-f. These numbers differ from the mean ± SD number of oocytes retrieved reported in Table 2. Furthermore, the magnitude of SD values reported in Supplementary Table 1 are within the expected range and in line with the values reported for the other RCTs used in our meta-analysis.

In conclusion, we believe that the correct data for mean ± SD for total number of oocytes retrieved are those reported in Supplementary Table 1 (15.4 ± 7.5 for Follitrope and 13.9 ± 6.4 for GONAL-f®), whereas the data reported in Table 2 (14.9 ± 0.5 for Follitrope and 12.8 ± 0.9 for GONAL-f) are actually the least square mean ± SE, as described in the Primary efficacy subsection [2]. Consequently, this will affect the results for the number of oocytes retrieved as presented in the Forest plot in our meta-analysis (Fig. 3B) [1]. The corrected Forest plot (Fig. 3B) is presented below.

**Fig. 3:**
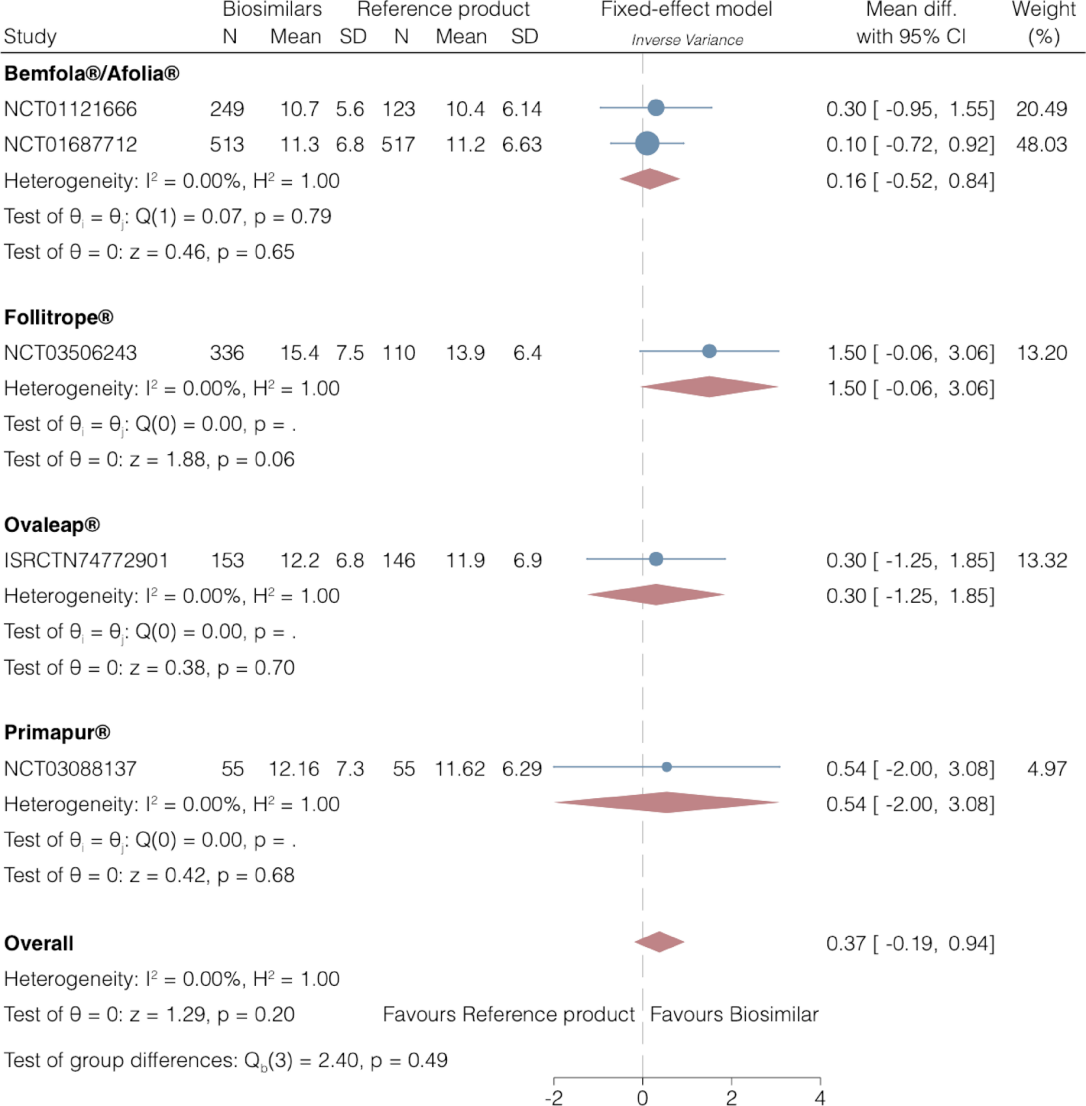
(Corrected Figure 3B) Mean difference number in of oocytes retrieved

This correction also necessitates the following amendments to the text of our meta-analysis [1]

In the Results (secondary endpoint) section, the following sentence “In addition, there was insufficient evidence for a difference in the total dose of gonadotrophins; however, a significantly higher number of oocytes was retrieved, and a significantly shorter duration of ovarian stimulation was observed with biosimilar preparations versus the reference product (Fig. 3).”

Should now read as “In addition, there was insufficient evidence for a difference in the total dose of gonadotrophins or the number of oocytes retrieved, while a significantly shorter duration of ovarian stimulation was observed with biosimilar preparations versus the reference product (Fig. 3).”

Furthermore, the following paragraph in the Discussion is no longer applicable and should be disregarded.

“Our findings show that although the number of oocytes retrieved was slightly higher (one more egg in all studies, except in the Follitrope^®^ study reporting two more eggs), lower pregnancy rates were reported with biosimilar preparations versus the reference product. To investigate this further, we conducted an additional analysis which excluded the Follitrope^®^ study [17], which was identified as having a high risk of bias. The exclusion of the Folitrope^®^ study from the analysis resulted in insufficient evidence for a difference in the number of oocytes retrieved with GONAL-f^®^ versus biosimilars (mean difference 0.20, 95% CI -0.41, 0.81; 4 RCTs; *n* =1881; I^2^ = 0%, moderate quality evidence). This finding should therefore be interpreted with caution. Furthermore, the mean total number of eggs varied between 10 and 15 in the five RCTs considered (Fig. 3b), which are normal numbers expected from a population with a normal ovarian reserve receiving a 150 – 225 IU r-hFSH starting dose [2, 50–52]. Therefore, this observation is not in conflict with current opinion that the number of oocytes retrieved positively correlates with downstream fertility treatment outcomes, including pregnancy and live birth [50–58].”

Finally, the following version of Supplementary Table 4 (in which the corrected values for the number of oocytes retrieved are highlighted) should be used to in place of the version published in the original article.

## Supplementary Information

**Additional file 1: Corrected Supplementary Table 4.** Outcomes of the randomised controlled trials included in the meta-analysis.
