# Supplementary material for: Biosimilar recombinant follitropin alfa preparations versus the reference product (Gonal-F®) in couples undergoing assisted reproductive technology treatment: a systematic review and meta-analysis
Source: Reprod Biol Endocrinol. 2021 Apr 2;19:51. doi: 10.1186/s12958-021-00727-y (PMC8017804; doi:10.1186/s12958-021-00727-y)
Supplement: Supplementary file 1 — Additional file 1. Supplementary Figure 1. Flow chart of study selection. Supplementary Figure 2. Relative risk for live birth rate with biosimilar preparations of follitropin alfa versus reference product (sensitivity analysis excluding the study with an unclear method of randomisation). Supplementary Table 1. Search strategy. Supplementary Table 2. Main characteristics of the randomised controlled trials included in the meta-analysis. Supplementary Table 3. Population characteristics, details of assisted reproductive technology treatment protocol used, outcomes evaluated and adjustment for confounders of the randomised controlled trials included in the meta-analysis. Supplementary Table 4. Outcomes of the randomised controlled trials included in the meta-analysis. Supplementary Table 5. Summary of the randomised controlled trials detected by search strategy without fertility outcomes. [file 12958_2021_727_MOESM1_ESM.docx]

Additional file 1: Supplementary (docx)

- Supplementary Figure 1. Flow chart of study selection
- Supplementary Figure 2. Relative risk for live birth rate with biosimilar preparations of follitropin alfa versus reference product (sensitivity analysis excluding the study with an unclear method of randomisation)
- Supplementary Table 1. Search strategy
- Supplementary Table 2. Main characteristics of the randomised controlled trials included in the meta-analysis
- Supplementary Table 3. Population characteristics, details of assisted reproductive technology treatment protocol used, outcomes evaluated and adjustment for confounders of the randomised controlled trials included in the meta-analysis
- Supplementary Table 4. Outcomes of the randomised controlled trials included in the meta-analysis
- Supplementary Table 5. Summary of the randomised controlled trials detected by search strategy without fertility outcomes

Supplementary materials

Supplementary Figure 1. Flow chart of study selection


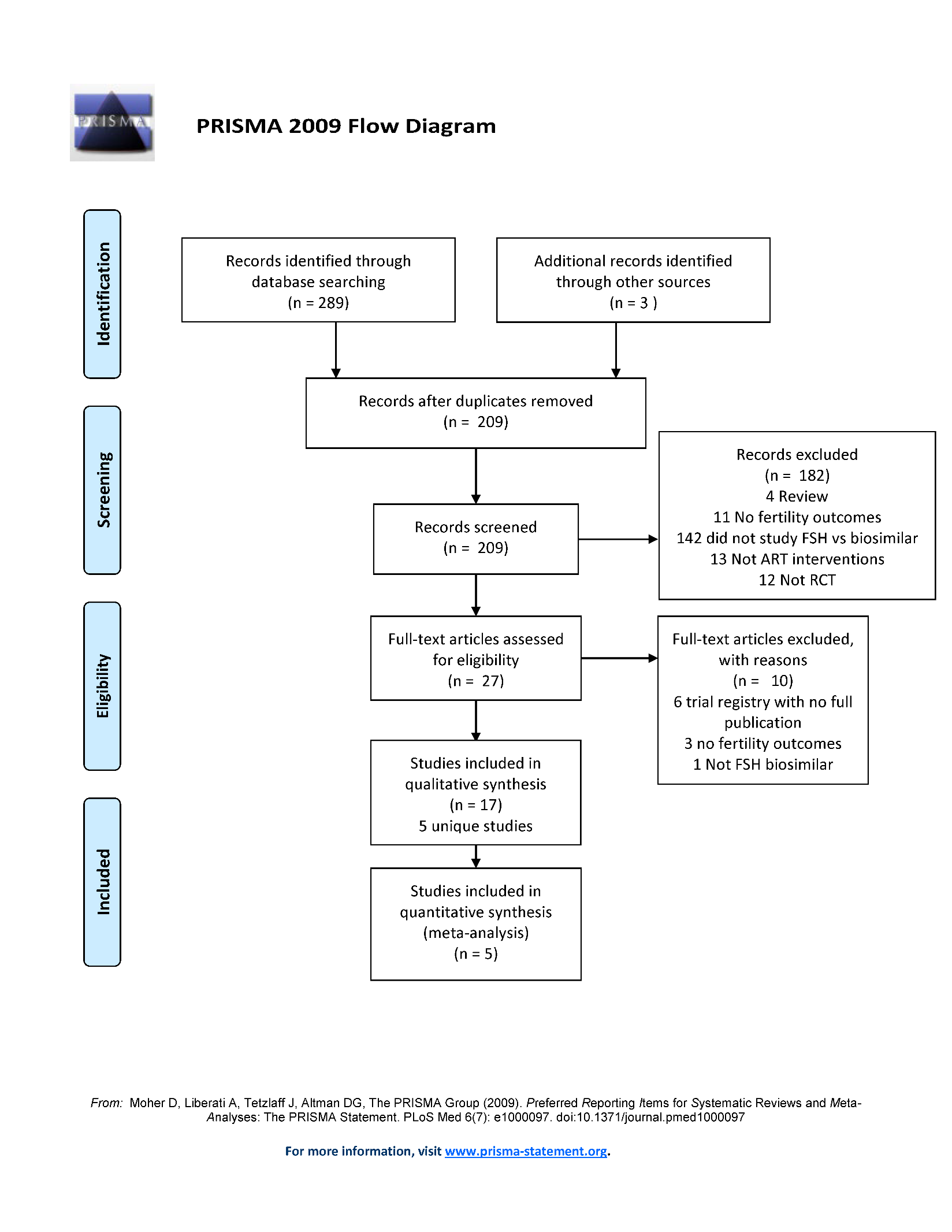


In the six registered trials (CTRI/2013/06/003749, 2013; CTRI/2016/06/006993, 2016; CTRI/2016/10/007367, 2016; EUCTR2013-003788-67-BE, 2013; IRCT2015110313907N2, 2016; NCT02454556, 2015) where data had not been published, the corresponding authors were contacted in an attempt to obtain data; however, no reply was received.

ART, assisted reproductive technology; RCT, randomised controlled trial.

Supplementary Figure 2. Relative risk for live birth rate with biosimilar preparations of follitropin alfa versus reference product (sensitivity analysis excluding the study with an unclear method of randomisation)

*
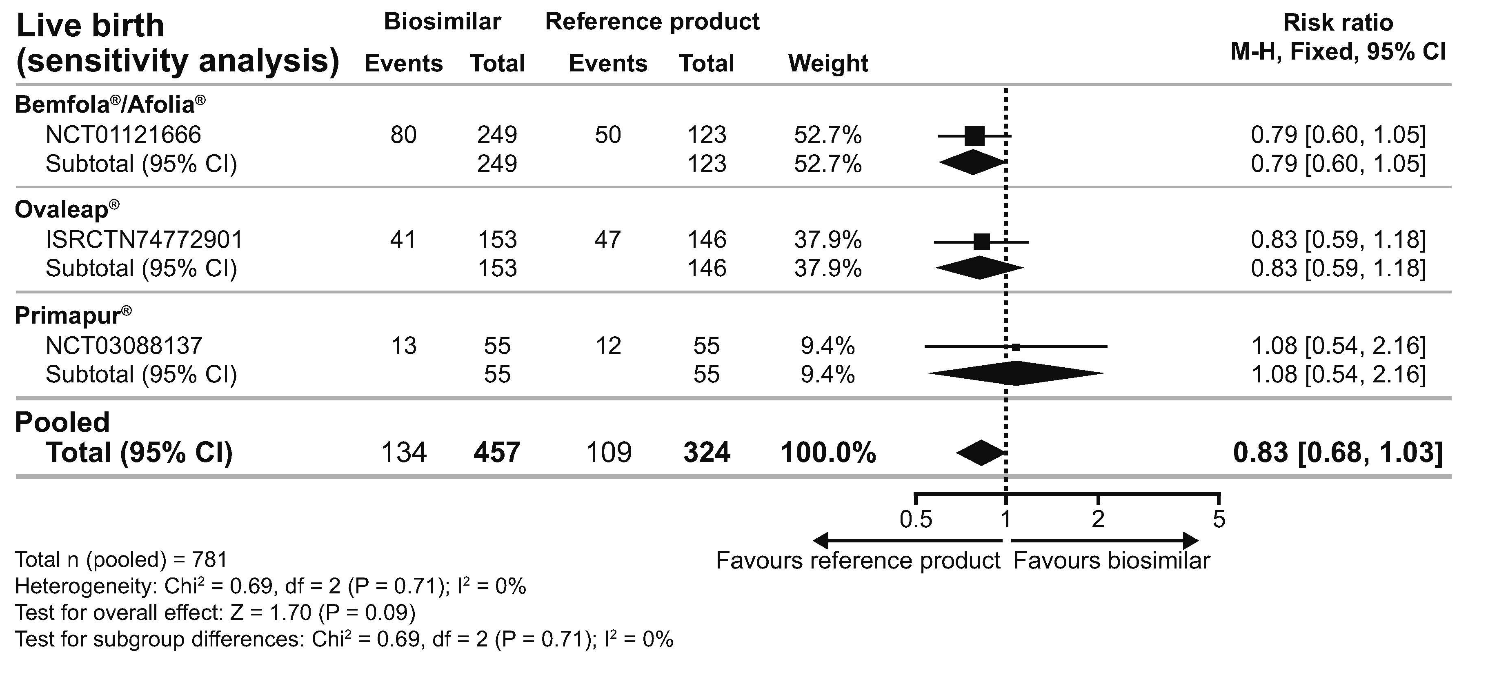
*

Supplementary Table 1. Search strategy

| Database  MEDLINE (ovid interface) |
| --- |
| Date  From inception until 29.8.2019 |
| Key words/terms   1. Gonal*.mp. 2. (Ovaleap* or XM17 or Bemfola*).mp. 3. (biosimilar* and (recombinant adj2 ("follicle stimulating hormone" or FSH or HFSH or follitropin))).mp. 4. Biosimilar Pharmaceuticals/ and (recombinant follicle stimulating hormone, human/ or ovulation induction/ or recombinant proteins/) 5. 2 or 3 or 4 6. 1 and 5 7. limit 6 to randomized controlled trial 8. (random* or factorial* or crossover* or cross over* or placebo* or (doubl* adj blind*) or (singl* adj blind*) or assign* or allocat* or volunteer*).mp. 9. 6 and 8 10. 7 or 9 |
| Database  Embase (ovid interface) |
| Date  From inception until 29.8.2019 |
| Key words/terms   1. Gonal*.mp. 2. (Ovaleap* or XM17 or Bemfola*).mp. 3. (biosimilar* and (recombinant adj2 ("follicle stimulating hormone" or FSH or HFSH or follitropin*))).mp. 4. biosimilar agent/ and recombinant follitropin/ 5. 2 or 3 or 4 6. 1 and 5 7. cm.fs. or comparative study/ or compar*.mp. 8. 6 and 7 9. crossover-procedure/ or double-blind procedure/ or randomized controlled trial/ or (random* or factorial* or crossover* or cross over* or placebo* or (doubl* adj blind*) or (singl* adj blind*) or assign* or allocat* or volunteer*).tw. 10. 8 and 9 |
| Database  Cochrane Library |
| Date  From inception until 29.8.2019 |
| Key words/terms   1. Gonal* 2. ovaleap* or XM17 or Bemfola 3. biosimilar 4. recombinant near/2 ("follicle stimulating hormone" or FSH or HFSH or follitropin*) 5. #3 AND #4 6. [mh "biosimilar pharmaceuticals"] 7. [mh "follicle stimulating hormone, human"] or [mh "ovulation induction"] or [mh "recombinant proteins"] 8. #6 AND #7 9. #2 OR #5 OR #8 10. #1 AND #9 11. random* or factorial* or crossover* or cross over* or placebo* or blind* or assign* or allocat* or volunteer* 12. #10 AND #11 |
| Database  Web of Science |
| Date  From inception until 29.8.2019 |
| Key words/terms   1. TS=Gonal* 2. TS=(ovaleap* or XM17 or Bemfola) 3. TS=biosimilar 4. TS=(recombinant NEAR/2 ("follicle stimulating hormone" or FSH or HFSH or follitropin*)) 5. #3 AND #4 6. #5 OR #2 7. #6 AND #1 8. TS=(random* or factorial* or crossover* or cross over* or placebo* or blind* or assign* or allocat* or volunteer*) 9. #8 AND #7 |
| Database  Clinicaltrials.gov |
| Date  From inception until 29.8.2019 |
| Key words/terms  Gonal |
| Database  WHO ICTRP |
| Date  From inception until 29.8.2019 |
| Key words/terms  Gonal |
| Database  EMA |
| Date  From inception until 29.8.2019 |
| Key words/terms  follitropin |
| Database  FDA |
| Date  From inception until 29.8.2019 |
| Key words/terms  follitropin |

Supplementary Table 2. Main characteristics of the randomised controlled trials included in the meta-analysis

| Clinical trial registration number | Country | Recruitment period | Multi-centre (Yes/No) | Sample size (reference product /\|biosimilars) | Power Analysis (Yes/No) | Method of randomisation | Blinding | Allocation concealment | Primary outcome measure assessed | Financial support | Authors contacted (Yes/No) |
| --- | --- | --- | --- | --- | --- | --- | --- | --- | --- | --- | --- |
| NCT01121666 (Bemfola/Afolia) | Multiple (EU) | July 2010 –April 2012 | Yes (15 centres, 6 countries) | 372 (123/249) | Yes | Computer algorithm | Single (investigator / outcomes assessor) | Via an interactive web or voice responses system | Number of cumulus-oophorus complexes | Sponsored by FINOX AG (currently Fertility Biotech) | No |
| ISRCTN74772901 (Ovaleap) | Multiple (EU) | March 2010 –July 2011 | Yes (20 centres, 5 countries) | 299 (146/153) | Yes | Computer algorithm | Single (investigator / outcomes assessor) | Via an interactive web or  voice responses system | Number of cumulus-oophorus complexes | Sponsored by Teva Branded Pharmaceutical Products R&D, Inc. | Yes |
| NCT01687712 (Bemfola/Afolia) | USA | 25 November 2013–10 September 2015 | Yes (22 centres) | 1101 (549/551) 1 not accounted for | Not reported | Not reported | Single (investigator / outcomes assessor) | Not reported | Clinical pregnancy 6 weeks post-embryo transfer | Sponsored by Fertility Biotech AG | Yes |
| NCT03088137 (Primapur) | Russia | 8 February 2017 –17 August 2018 | Yes (3 centres) | 110 (55/55) | Yes | Computer algorithm | Single (outcomes assessor) | Via an interactive web or  voice responses system | Number of cumulus-oophorus complexes | Sponsored by IVFarma LLC (Moscow, Russia) | No |
| NCT03506243  (Follitrope®) | China | April 2015 - May 2017 | Yes (6 centres) | 451 (112/339) | Yes | Computer algorithm | Single (investigator / outcomes assessor) | Sequentially numbered envelopes | Number of cumulus-oophorus complexes | Sponsored by Hangzhou Yuyuan Bioscience Technology Co., Ltd | No |

Supplementary Table 3. Population characteristics, details of assisted reproductive technology treatment protocol used, outcomes evaluated and adjustment for confounders of the randomised controlled trials included in the meta-analysis

| Clinical trial registration number | NCT01121666 (Bemfola^®^/Afolia) | ISRCTN74772901 (Ovaleap) | NCT01687712 (Bemfola/Afolia) | NCT03088137 (Primapur) | NCT03506243 (Follitrope^®^) |
| --- | --- | --- | --- | --- | --- |
| Inclusion criteria | Age 20-38 years; BMI 18-30 kg/m^2^; regular menstrual cycles of 25–35 days; 1st or 2nd cycle in the present series of assisted reproduction technique; basal FSH < 10 IU/L (cycle day 2–5); oestradiol levels < 50 pg/mL on the first day of FSH administration; antral follicle count of 10–25 follicles; infertility resulting from tubal factors; mild endometriosis ASRM stage 1–2; male factor; unexplained infertility; and presence of both ovaries and normal uterine cavity as confirmed by transvaginal ultrasound within 6 months before randomisation | Age 18 to 37 years; infertile; normogonadotrophic, 2 confirmed normal ovaries, undergoing controlled ovarian stimulation with ART therapy; regular menstrual cycles of 21-35 days; BMI 18-29 kg/m^2^; basal FSH, estradiol, prolactin, and thyroid-stimulating hormone concentrations in the normal range; able to give written informed consent | Age 35-42 years; Indication for IVF or ICSI; Regular menstrual cycles (25-35 days); History of a maximum of two fresh cycle treatments in the present series of ART at the day of first screening (thawed cycles are not subject to that criteria); BMI 18-38 kg/m^2^; Basal FSH <12 IU/L (cycle day 2-5); AFC 10-20 follicles with a diameter of <11mm in menstrual cycle day 2-5; infertility due to any of the following factors: tubal factor, mild endometriosis (ASRM stage 1-2), male factor, unexplained infertility; presence of both ovaries by ultrasonography and normal uterine cavity (confirmed by hysterosalpingography, saline infusion sonography or hysteroscopy within 6 months before randomisation); male partner with semen analysis that is at least adequate for ICSI within 6 months prior to patient beginning down-regulation (invasive or surgical sperm retrieval, donor and/or cryopreserved sperm may be used); willingness to participate in the study and to comply with the study protocol; signed informed consent prior to screening | Age 20–35 years old with a regular menstrual cycle (duration: 21–35 days, established causes of infertility: tubal and/or male factors, first or second attempt at IVF/ICSI; 18 BMI 30 kg/m^2^; FSH 10<IU/l and oestradiol level <50 pg/ml (cycle day 2–5); AMH >= 1.0 ng/ml; 4 AFC 15, written consent | Age 20-39 years; regular menstrual cycle (duration: 25–35 days; normal baseline FSH, LH, oestradiol and progesterone levels; indication for IVF; written consent |
| Exclusion criteria | >2 previous assisted reproduction  technique retrieval cycles; the presence of endocrine disorder; known tumours of the hypothalamus and pituitary gland, or both; a history of severe ovarian hyperstimulation syndrome; severe endometriosis (ASRM stage 3 or 4); the presence of a hydrosalpinx; polycystic  ovaries (Rotterdam criteria); history of poor response to gonadotrophin treatment (defined as <5 oocytes retrieved  in a previous attempt); and any hormonal treatment within 1 month before starting FSH treatment (with the exception  of levothyroxin) | >2 previously completed consecutive unsuccessful IVF cycles; > 3 miscarriages; history of severe OHSS; primary ovarian failure; categorised as poor responders to ovarian stimulation; hypersensitivity or allergy to recombinant FSH preparations; any significant cardiovascular, pulmonary, neurologic, endocrine, hepatic, or renal disease; neoplasm or a history of chemotherapy or radiation therapy; use of clomiphene or gonadotrophins within 30 days prior to enrolment. | Presence of pregnancy; PCOS; AFC >20 follicles with a diameter of <11 mm in menstrual cycle day 2-5; >2 previous unsuccessful fresh ART retrieval cycles; Uncontrolled endocrine disorder; Previous history or presence of severe OHSS; Intrauterine fibroids ≥5 cm or otherwise clinically relevant pathology that could impair embryo implantation or pregnancy continuation; History of recurrent spontaneous abortion (3 or more, even when unexplained); Presence of severe endometriosis (ASRM stage 3 or stage 4) or hydrosalpinx; Neoplasia, including tumors of the hypothalamus and pituitary gland; Abnormal bleeding of undetermined origin; History of extrauterine pregnancy in the previous 3 months; Known allergy or hypersensitivity to progesterone or to any of the excipients (including peanut oil) of the additional study medications (GnRH agonist, Ovidrel®, and Crinone 8%®); History of poor response to gonadotrophin treatment (defined as fewer than 5 oocytes retrieved in a previous attempt); Any hormonal treatment within 1 month before the start of the FSH treatment, with the exception of levothyroxine); Egg donor; Administration of other investigational products within the previous month; Clinically abnormal findings at Visit 1; Concomitant participation in another study protocol | Established contraindications to the use of ART methods; hypersensitivity to follitropin alfa or excipients; >=2 cycles of IVF/ICSI; history of severe OHSS; PCOS; endometriosis; uterine cavity pathology; history of poor or excessive response to stimulation with a r-hFSH; severe oligoasthenoteratozoospermia; azoospermia. | Clinically significant systemic/endocrine/metabolic disease; BMI >30 kg/m^2^; uterine cavity pathology; polycystic ovary; ovarian cyst; hydrosalpinx; previous uterine/ovarian surgery; previous OHSS; previous poor ovarian response; clomiphene or gonadotrophin within 1 month prior to randomisation; failure to reach standard follicles after 12-16 days of downregulation |
| Number of cycles | 2 cycles (no cross-over) | maximum of 3 cycles (crossover to exclusive use of Ovaleap in cycle 2 and 3) | maximum of 3 cycles | 1 cycle | 1 cycle |
| GnRH protocol used | Agonist (triptorelin or buserelin or leuprorelin) | Agonist (buserelin) | Not reported | Antagonist (ganirelix) | Agonist (triptorelin) |
| Type of follitropin alfa | Bemfola/Afolia and GONAL-f | Ovaleap and GONAL-f | Bemfola/Afolia and GONAL-f RFF | Primapur and GONAL-f | Follitrope and GONAL-f |
| Day of initiation | Successful downregulation (E2 level of less than 50 pg/mL, a shedded endometrium thickness < 5 mm and no ovarian cysts) | Successful downregulation (E2 <50 pg/ml, negative pregnancy test and no ovarian cysts >10mm) | Successful downregulation | Day 2-3 of menstrual cycle | Successful downregulation (median antral follicle size <10mm) |
| Starting dose (IU) | 150 | 150 | 225 | 150 | 150 -300 based on age |
| Dose adaption criteria | Adjusted after day 6 if risk of OHSS, decreased / coasting / terminated | Adjusted after day 5 to maximum 450 IU/day based serum oestradiol and ultrasound (every 2-3 days in increments of 37.5 IU, no more than 150IU) | Adjusted after day 5 to maximum 450 IU/day or minimum 75 IU/day (in increments of 37.5 IU) | Adjusted after day 5 to maximum 450 IU/day based on ultrasound | Adjusted after day 5 based on ovarian response |
| Criteria for hCG administration | ≥1 follicle >18mm and 2 follicles >16mm | ≥3 follicles ≥17 mm (E2 levels below 5500 pg/mL) | Not reported | Leading 2-3 follicles <18mm (if at risk of OHSS >15 follicles <14mm) | ≥1 follicles ≥18 mm / ≥3 follicles ≥16mm |
| Trigger type and dose | r-hCG (250µg) | r-hCG (dose not reported) | Not reported | hCG (5000 - 10000 IU) or GnRH agonist (0.2mg Decapeptyl) | hCG (5000 - 10000 IU) |
| Ovum pick-up | 34-36 hours | 34-37 hours | Not reported | <37 hours | 36-38 hours |
| IVF/ISCI | IVF/ICSI | Not reported | Not reported | IVF/ICSI | IVF/ICSI |
| Day of ET | Day 2-5 | Not reported | Not reported | Day 3 / 5 | Day 3 |
| Luteal phase support protocol (type/duration) | Intravaginal utrogestan 3 x 200mg / day from day of ET until confirmation of clinical pregnancy (5-6 weeks after oocyte retrieval) | According to investigators discretion | Not reported | According to investigators discretion | IM progesterone 60mg/day for 10 weeks after ET |

AFC, antral follicle count; AMH, anti-Müllerian hormone; ART, assisted reproductive technology; ASRM, American Society for Reproductive Medicine; E2, estradiol; ET, embryo transfer; FSH, follicle- stimulating hormone; GnRH, gonadotrophin-releasing hormone; hCG, human chorionic gonadotrophin; ICSI, intracytoplasmic sperm injection; IVF, *in vitro* fertilization; OHSS, ovarian hyperstimulation syndrome; PCOS, polycystic ovary syndrome.

Supplementary Table 4. Outcomes of the randomised controlled trials included in the meta-analysis

| Trial registration number | Cycle | Intervention | Intention to treat, N | Per protocol, N | Duration of Stimulation (days) | | | Total dose of gonadotrophins (IU) | | | Number of oocytes retrieved | | | OHSS (moderate to severe), N | Live birth, N | Ongoing pregnancy (10-12 weeks), N | Clinical pregnancy (5-8 weeks), N | Ectopic pregnancy, N | Multiple pregnancy, N |
| --- | --- | --- | --- | --- | --- | --- | --- | --- | --- | --- | --- | --- | --- | --- | --- | --- | --- | --- | --- |
|  |  |  |  |  | Mean | SD | N | Mean | SD | N | Mean | SD | N |  |  |  |  |  |  |
| NCT01121666 (Bemfola^®^/Afolia) | Cycle 1 | Bemfola/Afolia | 249 | 220 | 10.6 | 1.91 | 249 | 1555.7 | 293 | 249 | 10.7 | 5.62 | 249 | 24 | 80 | 84 | 90 |  |  |
|  |  | GONAL-f | 123 | 113 | 10.7 | 1.72 | 123 | 1569.2 | 259.2 | 123 | 10.4 | 6.14 | 123 | 6 | 50 | 51 | 55 |  |  |
|  | Cycle 2 | Bemfola/Afolia |  | 72 | 10.9 | 1.33 | 72 | 1612.3 | 217.67 | 72 | 10.4 | 4.2 | 72 |  | 22 | 22 | 25 |  |  |
|  |  | GONAL-f |  | 38 | 10.9 | 1.31 | 38 | 1604.9 | 216.61 | 38 | 10.1 | 5.3 | 38 |  | 9 | 9 | 10 |  |  |
| ISRCTN74772901 (Ovaleap) | Cycle 1 | Ovaleap | 153 | 152 | 9.3 | 1.8 | 153 | 1536 | 496 | 153 | 12.2 | 6.8 | 153 | 4 | 41 | 42 | 43 | 2 | 13 |
|  |  | GONAL-f | 146 | 145 | 9.7 | 1.6 | 146 | 1614 | 485 | 146 | 11.9 | 6.9 | 146 | 2 | 47 | 49 | 52 | 1 | 9 |
| NCT01687712 (Bemfola/Afolia) | Cycle 1 | Bemfola/Afolia | 549 | 486 | 10.8 | 1.72 | 549 | 3209.2 | 1008.05 | 549 | 11.3 | 6.76 | 513 | 7 | 101 |  | 114 | 5 |  |
|  |  | GONAL-f | 551 | 494 | 11 | 1.67 | 551 | 3343.6 | 1005.08 | 551 | 11.2 | 6.63 | 517 | 8 | 122 |  | 138 | 5 |  |
|  | Cycle 2 | Bemfola/Afolia | 109 |  |  |  | 107 |  |  | 107 |  |  | 107 | 0 | 16 |  | 17 | 0 |  |
|  |  | GONAL-f | 120 |  |  |  | 119 |  |  | 119 |  |  | 119 | 2 | 25 |  | 26 | 1 |  |
|  | Cycle 3 | Bemfola/Afolia | 28 |  |  |  | 27 |  |  | 27 |  |  | 27 | 0 | 4 |  | 5 | 1 |  |
|  |  | GONAL-f | 24 |  |  |  | 24 |  |  | 24 |  |  | 24 | 0 | 0 |  | 0 | 0 |  |
| NCT03088137 (Primapur) | Cycle 1 | Primapur | 55 | 49 | 9.75 | 1.08 | 55 | 1532.7 | 267.2 | 55 | 12.16 | 7.28 | 55 | 0** | 13 | 13 |  | 0 | 1 |
|  |  | GONAL-f | 55 | 49 | 9.73 | 1.03 | 55 | 1517.9 | 255.2 | 55 | 11.62 | 6.29 | 55 | 2** | 12 | 16 |  | 0 | 1 |
| NCT03506243  (Follitrope®) | Cycle 1 | Follitrope | 339 | 336 | 10.7 | 1.6 | 336 | 1945.3 | 635.7 | 336 | 14.9 | 0.5 | 336 | 4 |  | 82 | 103 |  |  |
|  |  | GONAL-f | 112 | 110 | 11.1 | 1.4 | 110 | 2020.2 | 562.7 | 110 | 12.8 | 0.9 | 110 | 5 |  | 34 | 41 |  |  |

OHSS, ovarian hyperstimulation syndrome.

**Severe OHSS, moderate OHSS not reported.

Supplementary Table 5. Summary of the randomised controlled trials detected by search strategy without fertility outcomes

| Trial registration number | Biosimilar | Randomisation / Blinding | Inclusion criteria | Exclusion criteria | **Primary outcomes** | **Secondary outcomes** |
| --- | --- | --- | --- | --- | --- | --- |
| CTRI/2013/ 06/003749, 2013 (n=120) | Newmon-R™ (150-225 IU/day commencing on mense day 2) | Computer generated randomisation, concealed by sequentially numbered, sealed, opaque envelopes / ng Open Label | Infertile women between 20-39 years of age; Basal FSH < 10 IU/L; regular menstrual cycles of 24- 35 days of duration; Antral size follicles > 5; Normal functional ovaries and uterus; Willing to give voluntary written consent | 1.Uterine myoma (fibroids) 2. Primary ovarian failure, uncontrollable hyperthyroidism or secondary adrenal insufficiency 3.Body Mass Index more than 30 kg/m^2^ 4.Subjects with previous history of ovarian hyperstimulation syndrome in the previous IVF cycles 5.More than 3 previously consecutive unsuccessful IVF cycles 6.Any significant systemic disease, endocrine or metabolic abnormalities 7.Tumors of the ovary, breast, uterus, hypothalamus or pituitary gland 8.Ovarian cysts or enlarged ovaries, not related to PCOD 9.HIV or syphilis positive subjects 10.History of alcohol or drug addiction 11.Subjects smoking more than 5 cigarettes per day 12.History of hypersensitivity to recombinant human FSH or other pharmaceutical excipients of this drug 13.Positive serum pregnancy test 14.Undiagnosed vaginal bleeding 15.Subjects unable to understand the objectives, methods, etc. of this clinical study and are unable to comply with the study procedures 16.Participation in any other clinical trial within 3 months of registering in this study | Total number of oocytes retrieved | - Total administration dosage of recombinant human FSH - Duration of recombinant human FSH stimulation - E2 concentration on the day of hCG administration - Number of follicles more than 16mm in diameter on the day of hCG administration - Number of follicles more than or equal to 14 mm on the day of hCG - Number of oocytes fertilised and fertilization rate - Number of embryos transferred - Embryo implantation rate - Clinical pregnancy rate - Incidence rate of OHSS - Compliance score - Patient assessment for use |
| CTRI/2016/ 06/006993, 2016 (n=214) | Foligraf™  (fixed dose of 225 IU/day, SC, daily, for 5 days) | Computer generated randomisation / outcome assessor blinded | Age 20-39 year; indication for ART using COS; regular menstrual cycle of 21-35 days (both inclusive); normal FSH level; normal estradiol levels; good AFC; normal body mass index; results of clinical laboratory tests (haematology, blood chemistry, and urinalysis) within normal reference range, or outside the reference range; willing to provide written informed consent and comply with the study procedures | 1. History of successive ART cycles 2. History of any endocrine abnormality 3. History of ovarian hyperresponse 4. Polycystic ovarian syndrome 5. One ovary or ovarian abnormality 6. Severe endometriosis 7. Submucosal fibroids or any other clinically relevant pathology, which could impair embryo implantation or pregnancy continuation 8. History of extrauterine pregnancy 9. History of poor response to gonadotrophin treatment in the previous ART cycle; 10. History of miscarriages 11. Tested positive for HIV, Hepatitis B or Hepatitis C at screening; 12. Allergic, hypersensitive, or intolerant to any of the preparations of recombinant human FSH 13. Contraindications to the use of gonadotrophins 14. History of epilepsy, thrombophilia, cardiovascular, gastro-intestinal, hepatic, renal, pulmonary, auto-immune disease or any active infection, requiring treatment which at the investigator’s discretion might interfere with the study; 15. History of malignancy; 16. Smokes or has stopped smoking within the last 3 months prior to screening; 17. History of alcohol or drug abuse 18. Any treatment that can have teratogenic effect or affect ovulation 19. Received an investigational product or has used an invasive investigational medical device within 30 days before the planned first dose of the study drug; or is currently enrolled in an investigational study. | Total number of oocytes retrieved | - Immunogenicity - Pregnancy rate - Number of oocytes retrieved in <35 years vs >=35 years - Cycle cancellation - Total dose recombinant FSH - Duration of stimulation - Dose adjustment required (%) - Endocrinological parameters - Enodmetrial thickness - Quality of oocytes / embryos - Dose reduction due to imminent OHSS - Any adverse event reported |
| CTRI/2016/ 10/007367, 2016 (n=116) | Recombinant FSH Cadila Healthcare Limited, Indi | Computer generated randomisation concealed with centralised system / open label | 1. Females between 22-38 years of age with regular menstrual cycle of 24-35 days 2. Infertile female patients undergoing COH for ART 3. BMI between 18-30 kg/m^2^ inclusive page 4 / 6 PDF of Trial CTRI Website URL - http://ctri.nic.in 4. Transvaginal ultrasound documenting the presence of both ovaries without abnormalities and normal uterine adnexa 5. Clinically acceptable ranges of Basal FSH, LH, E2 at the time of enrolment 6. AFC 8 to 25 follicles (sum of both ovaries) 7. Willing to comply with all the study requirements and procedures 8. Normal or clinically insignificant haematology, serum chemistry and urinalysis parameters during screening 9. Willing to provide written informed consent | 1. History of allergy or hypersensitivity reactions to FSH or any other ingredients of the formulation 2. Use of any FSH preparation or clomiphene citrate within 60 days of randomisation 3. History of > 2 succeeding ART retrieval cycles (which includes fresh and frozen embryo transfers before the study cycle without clinical pregnancy 4. Presence of PCOS 5. Previous history of severe ovarian hyperstimulation syndrome 6. Presence of severe endometriosis (ASRM stage 3 or stage 4) and hydrosalpinx 7. Presence or history of thrombophlebitis or thromboembolic disorders 8. History of extrauterine pregnancy in the previous 3 months 9. History of poor response to gonadotrophin treatment (defined as fewer than 5 oocytes retrieved in a previous attempt) 10. Subjects with clinically significant unstable medical disorders, life-threatening disease, or current malignancies. 11. Positive Pap Smear at screening 12. combination or hormonal implants 6 months prior to screening 13. Positive Pregnancy test at screening | Total number of oocytes retrieved | - Biochemical pregnancy rate after 2 weeks of embryo transfer - Total dose of recombinant human FSH require   Number of days of recombinant human FSH stimulation   - Number and size distribution of follicles at the day of ovulation induction - Percentage of patients with need to increase or lower the dose of recombinant human FSH - Number of good quality oocytes |
| EUCTR2013-003788-67-BE, 2013 (n=471) | Actavis recombinant human FSH | Not reported / outcomes assessor blinded | Age 18 to 35 years; BMI of 18 – 32; physical examination, including vital signs, that is within normal limit; serum FSH level less than 2x the upper range of normal on cycle day 3; tubal factor, ovulation disorder, mild endometriosis ASRM stage 1-2), male factor, other causes, unexplained cause, multiple factors (female only), and multiple factors (male and female); both ovaries present; normal uterine cavity, donor sperm allowed, sperm quality adequate for ICSI, no endocrine disorders, able to provide consent | Previous ART, another clinical trial, other hormonal treatments within 30 days, >=12 follicles in ovaria and/or ovarian volume >10ml, abnormal vaginal bleeding, symptoms of pelvic infection, endocrinological disorders, recurrent miscarriage, allergy to study drug, smoker, ectopic pregnancy in past 6 months | Total number of oocytes retrieved | - Ongoing pregnancy rate, defined as percentage of subjects with presence of at least one fetus with heart activity, at 10 weeks after embryo transfer; - Mean total dose of Actavis recombinant human FSH or GONAL-f required during the ART cycle - Mean number of days of Actavis recombinant human FSH or GONAL-f stimulation during the ART cycle; - Percentage of subjects who require an increase or decrease of the dose of Actavis recombinant human FSH or GONAL-f during treatment; - Mean number of follicles/cysts with sizes = 11 mm, = 15 mm, or = 17 mm on Treatment Day 6 (before FSH dose adjustment) and the day of hCG administration; - Serum concentrations of inhibin-B, E2, LH, FSH, and progesterone on Treatment Day 1 (before Actavis rhFSH or GONAL-f injection) and the day of hCG administration - Mean number of fertilised oocytes at 24 hours after the initiation of   the IVF for subjects who are not choosing ICSI procedure   - Mean number of metaphase II oocytes after oocyte retrieval for subjects with ICSI procedure (metaphase II oocytes are defined as having extruded the first polar body and are in the resting phase of meiosis II); - Mean number of Grade 1 and Grade 2 embryos on Day 3 of embryo culture. |
| IRCT201511 0313907N2, 2016 (n=142) | Cinnal-F | Not randomised / double blinded | FSH <10; normal prolactinemia; no pathology in uterus and adenexae; regular menses between 25-35 days; not more than three times ICSI in history; variable factors of infertility | systemic diseases; BMI>30; history of severe OHSS; Hx. of poor response in previous cycles; any pathology in uterus and anenexae | Number of MII oocytes | Number of grade A embryos |
| NCT0245 4556, 2015 (n=106) | FOLITIME^®^ (225 IU / day from cycle day 2/3) | Randomised / outcomes assessor blinded | 1. 18 to 38 years of age  2. Indication for controlled ovarian stimulation and IVF or ICSI  3. Regular menstrual cycles (25-35 days)  4. History of a maximum of two fresh cycle treatments in the present series of ART at the day of first screening (thawed cycles are not subject to that criteria)  5. BMI ≥18 and ≤32 kg/m^2^  6. Basal FSH <10 IU/L (cycle day 2-5)  7. AFC ≥8 to ≤18 follicles with a diameter of <10mm (sum of both ovaries) as measured on ultrasound in the early follicular phase (day 1 of the stimulation cycle or of the last cycle previous to the treatment)  8. Documented history of infertility due to any of the following factors: tubal factor, male factor, unexplained infertility  9. Presence of both ovaries by ultrasonography and normal uterine cavity or abnormal uterine cavity without clinical significance according to the investigator's opinion (confirmed by hysterosalpingography, saline infusion sonography or hysteroscopy within 12 months before randomisation) and normal uterine cavity by transvaginal ultrasound within 3 months of treatment  10. Male partner with semen analysis that is at least adequate for ICSI within 6 months prior to patient beginning down-regulation (invasive or surgical sperm retrieval, donor and/or cryopreserved sperm may be used)  11. Willingness to participate in the study and to comply with the study protocol  Signed informed consent prior to screening | 1. Presence of pregnancy  2. History of or active PCOS  3. AFC >18 follicles with a diameter of <10 mm (both ovaries combined) as measured on US in the early follicular phase (menstrual cycle day 2-5) or during  4. History of >2 unsuccessful fresh ART retrieval cycles  5. History of poor response to gonadotrophin treatment (defined as fewer than 5 oocytes retrieved in a previous attempt)  6. Any hormonal treatment within 1 month before the start of the FSH treatment, with the exception of levothyroxine)  7. Egg donor  8. Intrauterine leiomyomas ≥5 cm or otherwise clinically relevant pathology that could impair embryo implantation or pregnancy continuation  9. Previous history of OHSS  10. Ovarian cyst or enlargement of undetermined origin  11. History of recurrent spontaneous abortion (3 or more, even when unexplained)  12. Presence of endometriosis or hydrosalpinx  13. Neoplasia, including tumors of the hypothalamus and pituitary gland  14. Abnormal genital bleeding of undetermined origin  15. History of extrauterine pregnancy in the previous 3 months  16. Sex hormone dependent tumors of the reproductive tract and accessory organs.  17. Uncontrolled thyroid or adrenal dysfunction or presence of uncontrolled endocrine disorder  18. Known allergy or hypersensitivity to FSH preparations or one of their excipients or progesterone or to any of the excipients of the additional study medications  19. Clinically significant abnormal findings at Visit 1 that, in the opinion of the investigator, can affect trial results or subject's safety  20. Administration of other investigational products within the previous month or concomitant participation in another study protocol | Number of oocytes retrieved | - Total dose of recombinant human FSH required, - Number of days of recombinant human FSH stimulation - Percentage of patients with need to increase or lower the dose of recombinant human FSH, - Number of treatment cycle cancellations and their reason - Fertilization rate - Number of fertilised oocytes - Number of good quality embryos - Number of embryos transferred - Implantation Rate - Biochemical pregnancy - Clinical pregnancy 10 weeks post embryo transfer - Pregnancy outcome - Safety - Incidence of - OHSS (and its severity) - Local reactions (pain, bruising, redness, itching, swelling) - Systemic drug adverse events - Tolerability - Frequency of patients who withdraw the study drug due to lack of tolerance - Frequency of patients who withdraw the study drug treatment due to any reason - Patient Reported Pain: measured by a Patients Visual Analog Scale - Immunogenicity Measurement of possible antibodies against exogenous recombinant human FSH will be evaluated. Pharmacodynamics - Number and size distribution of follicles during treatment - Number and size distribution of follicles at the day of ovulation induction (uurinary hCG) - Number of follicles >14 mm on the day of hCG injection. - Hormone parameters: serum levels of estradiol, luteinising hormone and progesterone on the day of hCG injection - Metaphase II oocytes; - Number of good quality oocytes |

AFC, antral follicle count; AMH, anti-Müllerian hormone; ART, assisted reproductive technology; ASRM, American Society for Reproductive Medicine; COH, controlled ovarian hyperstimulation; COS, controlled ovarian stimulation; E2, estradiol; FSH, follicle-stimulating hormone; GnRH, gonadotrophin-releasing hormone; hCG, human chorionic gonadotrophin; HIV, human immunodeficiency virus; ICSI, intracytoplasmic sperm injection; IVF, *in vitro* fertilization; LH, luteinizing hormone; OHSS, ovarian hyperstimulation syndrome; PCOD, polycystic ovarian disease; PCOS, polycystic ovary syndrome; PGD, pre-implantation genetic diagnosis.
