# Supplementary material for: Correction: Biosimilar recombinant follitropin alfa preparations versus the reference product (Gonal-F®) in couples undergoing assisted reproductive technology treatment: a systematic review and meta-analysis
Source: Reprod Biol Endocrinol. 2023 Jul 26;21:68. doi: 10.1186/s12958-023-01114-5 (PMC10369731; doi:10.1186/s12958-023-01114-5)
Supplement: Supplementary file 1 — Additional file 1: Corrected Supplementary Table 4. Outcomes of the randomised controlled trials included in the meta-analysis. [file 12958_2023_1114_MOESM1_ESM.docx]

Corrected Supplementary Table 4. Outcomes of the randomised controlled trials included in the meta-analysis.

| Trial registration number | Cycle | Intervention | Intention to treat, N | Per protocol, N | Duration of Stimulation (days) | | | Total dose of gonadotrophins (IU) | | | Number of oocytes retrieved | | | OHSS (moderate to severe), N | Live birth, N | Ongoing pregnancy (10-12 weeks), N | Clinical pregnancy (5-8 weeks), N | Ectopic pregnancy, N | Multiple pregnancy, N |
| --- | --- | --- | --- | --- | --- | --- | --- | --- | --- | --- | --- | --- | --- | --- | --- | --- | --- | --- | --- |
|  |  |  |  |  | Mean | SD | N | Mean | SD | N | Mean | SD | N |  |  |  |  |  |  |
| NCT01121666 (Bemfola^®^/ Afolia) | Cycle 1 | Bemfola/  Afolia | 249 | 220 | 10.6 | 1.91 | 249 | 1555.7 | 293 | 249 | 10.7 | 5.62 | 249 | 24 | 80 | 84 | 90 |  |  |
|  |  | GONAL-f | 123 | 113 | 10.7 | 1.72 | 123 | 1569.2 | 259.2 | 123 | 10.4 | 6.14 | 123 | 6 | 50 | 51 | 55 |  |  |
|  | Cycle 2 | Bemfola/  Afolia |  | 72 | 10.9 | 1.33 | 72 | 1612.3 | 217.67 | 72 | 10.4 | 4.2 | 72 |  | 22 | 22 | 25 |  |  |
|  |  | GONAL-f |  | 38 | 10.9 | 1.31 | 38 | 1604.9 | 216.61 | 38 | 10.1 | 5.3 | 38 |  | 9 | 9 | 10 |  |  |
| ISRCTN74772901 (Ovaleap) | Cycle 1 | Ovaleap | 153 | 152 | 9.3 | 1.8 | 153 | 1536 | 496 | 153 | 12.2 | 6.8 | 153 | 4 | 41 | 42 | 43 | 2 | 13 |
|  |  | GONAL-f | 146 | 145 | 9.7 | 1.6 | 146 | 1614 | 485 | 146 | 11.9 | 6.9 | 146 | 2 | 47 | 49 | 52 | 1 | 9 |
| NCT01687712 (Bemfola/ Afolia) | Cycle 1 | Bemfola/  Afolia | 549 | 486 | 10.8 | 1.72 | 549 | 3209.2 | 1008.05 | 549 | 11.3 | 6.76 | 513 | 7 | 101 |  | 114 | 5 |  |
|  |  | GONAL-f | 551 | 494 | 11 | 1.67 | 551 | 3343.6 | 1005.08 | 551 | 11.2 | 6.63 | 517 | 8 | 122 |  | 138 | 5 |  |
|  | Cycle 2 | Bemfola/  Afolia | 109 |  |  |  | 107 |  |  | 107 |  |  | 107 | 0 | 16 |  | 17 | 0 |  |
|  |  | GONAL-f | 120 |  |  |  | 119 |  |  | 119 |  |  | 119 | 2 | 25 |  | 26 | 1 |  |
|  | Cycle 3 | Bemfola/  Afolia | 28 |  |  |  | 27 |  |  | 27 |  |  | 27 | 0 | 4 |  | 5 | 1 |  |
|  |  | GONAL-f | 24 |  |  |  | 24 |  |  | 24 |  |  | 24 | 0 | 0 |  | 0 | 0 |  |
| NCT03088137 (Primapur) | Cycle 1 | Primapur | 55 | 49 | 9.75 | 1.08 | 55 | 1532.7 | 267.2 | 55 | 12.16 | 7.28 | 55 | 0** | 13 | 13 |  | 0 | 1 |
|  |  | GONAL-f | 55 | 49 | 9.73 | 1.03 | 55 | 1517.9 | 255.2 | 55 | 11.62 | 6.29 | 55 | 2** | 12 | 16 |  | 0 | 1 |
| NCT03506243  (Follitrope®) | Cycle 1 | Follitrope | 339 | 336 | 10.7 | 1.6 | 336 | 1945.3 | 635.7 | 336 | 15.4 | 7.5 | 336 | 4 |  | 82 | 103 |  |  |
|  |  | GONAL-f | 112 | 110 | 11.1 | 1.4 | 110 | 2020.2 | 562.7 | 110 | 13.9 | 6.4 | 110 | 5 |  | 34 | 41 |  |  |
